# Genetic remodeling of soil diazotrophs enables partial replacement of synthetic nitrogen fertilizer with biological nitrogen fixation in maize

**DOI:** 10.1038/s41598-024-78243-3

**Published:** 2024-11-12

**Authors:** Rafael Martinez-Feria, Maegen B. Simmonds, Bilge Ozaydin, Stacey Lewis, Allison Schwartz, Alex Pluchino, Megan McKellar, Shayin S. Gottlieb, Tasha Kayatsky, Richelle Vital, Sharon E. Mehlman, Zoe Caron, Nicholas R. Colaianni, Jean-Michel Ané, Junko Maeda, Valentina Infante, Bjorn H. Karlsson, Caitlin McLimans, Tony Vyn, Brendan Hanson, Garrett Verhagen, Clayton Nevins, Lori Reese, Paul Otyama, Alice Robinson, Timothy Learmonth, Christine M. F. Miller, Keira Havens, Alvin Tamsir, Karsten Temme

**Affiliations:** 1 Pivot Bio, Inc., 2910 Seventh St, Berkeley, CA 94710 USA; 2 Department of Plant and Agroecosystem Sciences, University of Wisconsin-Madison, 1575 Linden Drive, Madison, WI 53706 USA; 3 Department of Agronomy, Purdue University, 915 Mitch Daniels Blvd, West Lafayette, IN 479074 USA; 4 Present Address: Regrow Agriculture, Inc. , Durham , NH 03824 USA

**Keywords:** Synthetic biology, Agroecology

## Abstract

Increasing biological nitrogen (N) fixation (BNF) in maize production could reduce the environmental impacts of N fertilizer use, but reactive N in the rhizosphere of maize limits the BNF process. Using non-transgenic methods, we developed gene-edited strains of *Klebsiella variicola* (*Kv*137-2253) and *Kosakonia sacchari* (*Ks*6-5687) bacteria optimized for root-associated BNF and ammonium excretion in N-rich conditions. The aim of this research was to elucidate the mechanism of action of these strains. We present evidence from in vitro, in planta and field experiments that confirms that our genetic remodeling strategy derepresses BNF activity in N-rich systems and increases ammonium excretion by orders of magnitude above the respective wildtype strains. BNF is demonstrated in controlled environments by the transfer of labeled ^15^N_2_ gas from the rhizosphere to the chlorophyll of inoculated maize plants. This was corroborated in several ^15^N isotope tracer field experiments where inoculation with the formulated, commercial-grade product derived from the gene-edited strains (PIVOT BIO PROVEN® 40) provided on average 21 kg N ha^-1^ to the plant by the VT-R1 growth stages. Data from small-plot and on-farm trials suggest that this technology can improve crop N status pre-flowering and has potential to mitigate the risk of yield loss associated with a reduction in synthetic N fertilizer inputs.

## Introduction

Synthetic N fertilizers derived from the Haber–Bosch fixation of dinitrogen gas (N_2_) are credited to have enabled the sustenance for about half of the world’s population^1^, contributing circa 48% of the N harvested in major cereal crops worldwide^2–5^. However, this benefit has come with steep environmental costs. This is because only a portion of applied synthetic N fertilizer is typically recovered by cereal crops^6,7^, while the excess reactive N is prone to loss from agricultural soils. Nitrate (NO_3_) leachate pollutes freshwater sources and marine ecosystems^7,8^, and soil nitrous oxide (N_2_O) emissions are an important contributor to stratospheric ozone depletion^9^and a potent greenhouse gas (GHG)^10^. Soil N_2_O emissions associated with N fertilizer applications, along with the embedded GHG emissions from their manufacturing, transport and application, collectively account for nearly 2.5% of global GHG emissions^11^.

Reducing synthetic N fertilizer use is considered key to effectively reducing NO_3_ leaching and N_2_O emissions^11,12^. However, doing so without negatively impacting cereal yields is challenging, as it requires tight synchronization between N supply and crop N demand to achieve sustainable N fertilizer use efficiency^13–15^. Various strategies have been proposed to accomplish this, including fine tuning crop N supply by improving fertilizer timing, rate, source, and placement^16^; optimizing the timing of crop N demand via higher planting density, earlier planting dates, and crop genetic improvement^17^; and enhancing soil mineral N retention for subsequent crop uptake with crop rotations, cover crops, reduced tillage, and drainage management^18^. While several of these strategies have been shown to improve N fertilizer use efficiency^13^, there remain critical socio-economic barriers to grower adoption^13,19^. Consequently, the United States (U.S.) and other industrialized countries have made little progress towards reducing N fertilizer consumption^2,13,20^. Therefore, there is a need for innovative solutions that enable sustainable reductions in N fertilizer use to abate its environmental impacts without compromising crop productivity.

Biological N fixation (BNF) is one of the dominant pathways for acquiring newly available N in most terrestrial ecosystems and is a major supplier of N in legume crops^21,22^. Although cereals lack the specialized structures needed to form symbiotic relationships, BNF performed by free-living, associative, and endophytic diazotrophs can still make significant contributions to cereal N uptake in some conditions^23^; nearly a quarter of the N harvested globally in maize, wheat and rice is estimated to have originated from BNF from 1961–2010^5^. However, nitrogenase, the main enzyme responsible for the reduction of N_2_to ammonia, is downregulated by the presence of reactive N^21^, such that N fertilizer application greatly reduces the contribution of BNF to cereal N uptake^24^. Thus, BNF has not been compatible with modern cereal production that is heavily reliant on N fertilizers. Augmenting BNF in these conditions could diminish our reliance on energy- and GHG-intensive synthetic N sources. Additionally, because BNF performed by root-associated or endophytic diazotrophs has the potential to better match crop N uptake demand spatially and temporally compared to other sources of N^22,25^, the risk of environmental loss of reactive N could be mitigated.

Re-engineering diazotrophs’ genetic constraints can enhance BNF under high N fertility conditions^25–27^. Previous research^27^ demonstrated the successful genetic remodeling of the root-associated diazotroph *Klebsiella variicola* (*Kv*137-1036) isolated from field-grown maize. Removing the repression of nitrogenase in N-rich environments (i.e., derepression) in its parent strain, *Kv*137, resulted in > 100-fold increase in nitrogenase activity in vitro in N-rich media^27^. Furthermore, *Kv*137-1036 exhibited colonization levels similar to *Kv*137 on the roots of three-week-old maize plants while displaying higher BNF activity in the rhizosphere. The commercial utility of the formulated product (PIVOT BIO PROVEN®) was validated for biosafety, product stability, and an average grain yield boost of 0.3 $\pm$ 0.1 Mg ha^-1^. Additionally, there was an 8 to 25% reduction in within-field yield variability relative to an uninoculated control in multiyear and multisite commercial-scale on-farm field trials^27^. Although *Kv*137-1036 was confirmed to excrete ammonium (NH_4_^+^) due to passive excretion driven by higher rates of BNF^27^, further increases in NH_4_^+^ excretion may be possible with targeted edits to downregulate N assimilation, and thereby increase N efflux into the maize rhizosphere. Further in-vitro research with a lineage of *Kosakonia sacchari* (*Ks*6) showed robust colonization of the maize rhizosphere across diverse field conditions and elevated levels of BNF in the presence of exogenous N with remodeled strains^26^, providing additional avenues for increasing BNF in fertilized cereal crops.

**Fig. 1:**
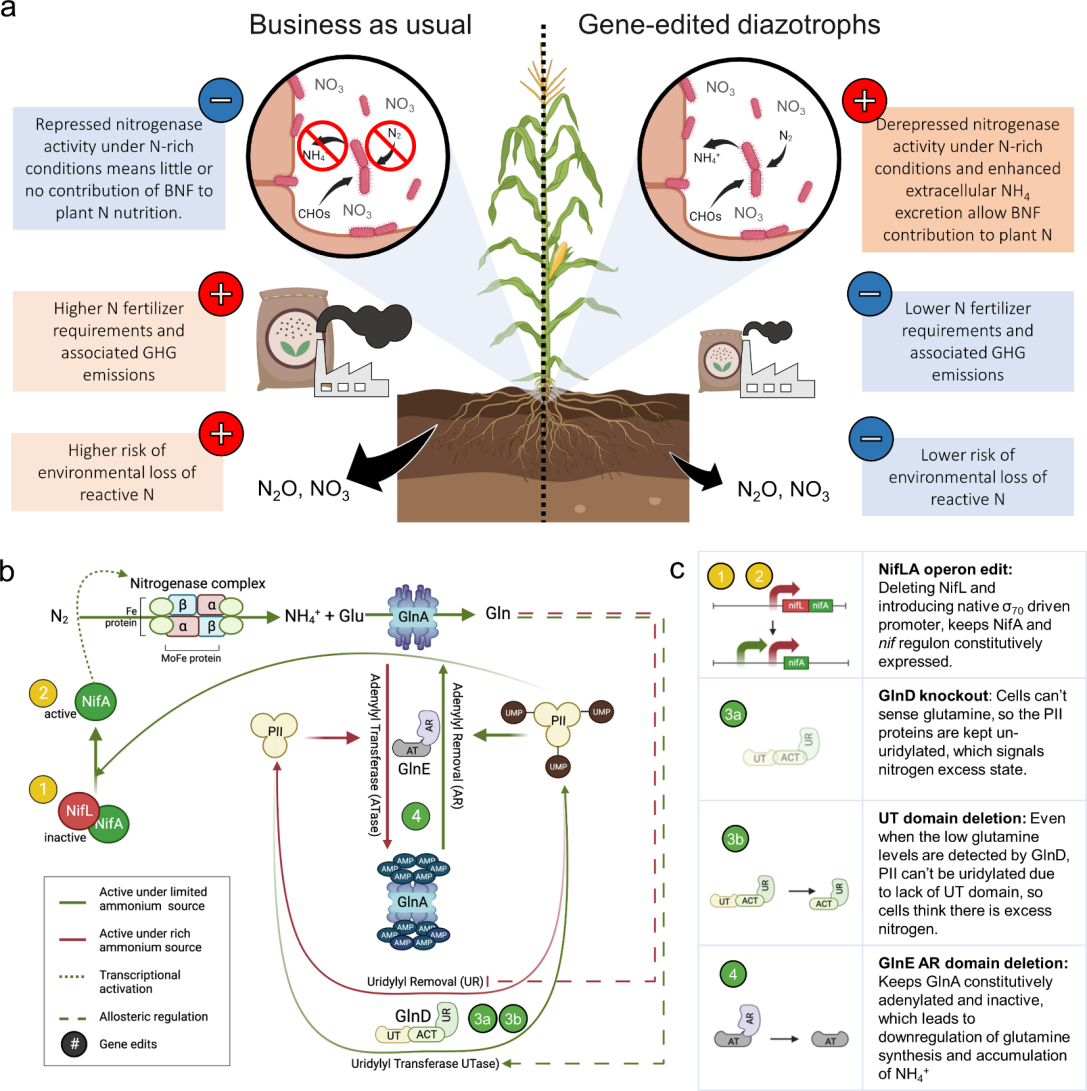
Redesigning maize nitrogen (N) nutrition with genetically remodeled root-associated soil diazotrophs for biological N fixation (BNF) under N-rich conditions.** (a)** Conceptual diagram of expected changes to N cycling in maize cropping systems resulting from better synchronization of crop N demand and N supply, and reduction in synthetic N requirements, and associated environmental N losses. **(b)** BNF and its regulatory pathways. NifA is the transcriptional activator of the nitrogenase complex and its accessory genes, collectively named *nif regulon*. Under abundant N sources, NifL protein inhibits NifA’s transcriptional activity. This inhibition is alleviated when low glutamine levels activate the Urydylyl-transferase activity of GlnD, the glutamine-sensing bifunctional protein (dashed green lines). GlnD, in turn, urydylylates PII signaling proteins, which represses the inhibitory effect of NifL and increases the expression of *nif*A (green arrows). Active NifA leads to the expression of *nif* regulon and BNF. The uridylated PII proteins also activate the adenylyl removal function of the GlnE protein, which in turn activates the glutamine synthetase (GlnA) activity for the assimilation of the fixed N via glutamine. Increased glutamine levels trigger the uridylyl removal activity of GlnD, which removes the uridylyl groups from the PII signaling proteins. In this study, we have edited four gene targets (numbered circles) to bypass the regulations and enable constitutive BNF (yellow circles) and ammonium excretion (green circles) in the diazotrophic strains. **(c)** Description of the derepression and excretion edits. To constitutively express *nifA*, we have deleted the negative regulator, *nifL* (1), and introduced a strong constitutive promoter upstream of the *nifA* gene (2). To decrease assimilation and increase ammonium excretion, we deleted the whole *glnD* gene in Ks6-5687 (3a) or removed its *uridylyl transferase (UT)* domain in Kv137-2253 (3b). Lastly, to further downregulate the assimilation of ammonium via glutamine synthetase (GlnA), we inactivated this protein by removing the adenylyl removal domain of the *glnE* gene, which kept GlnA in the inactive, adenylated state (4).

Although the N-fixation potential of these genetically remodeled strains under N-rich conditions has been confirmed in vitro^26,27^, and maize grain yield advantages have been observed in the case of *Kv*137-1036^27^, direct validation of plant-associated BNF (i.e., crop assimilation of fixed N_2_) is still needed. Furthermore, a deeper understanding of how inoculation affects maize plant N status and productivity can provide us with a roadmap for decoupling synthetic N fertilizer use from cereal grain production (Fig. 1a). The aim of this research was to elucidate BNF as the mechanism of action of two genetically remodeled strains (Fig. 1b-c), *Kv*137-2253 and *Ks*6-5687 (previously referred to as PBC6.99 in *ref.*^26^) for improving N supply and productivity in maize. (Details on the genetic remodeling strategy pursued to engineer these strains is provided on Fig. 1b-c and supplemental information text S1.) To do this, we used data from in vitro, in planta, and field experiments, and evaluated the response of maize plants to BNF, as well as the potential of the formulated, commercially available inoculant (PIVOT BIO PROVEN® 40, hereafter referred to as PROVEN 40) to replace a portion of synthetic N fertilizer inputs. Establishing evidence for the latter has tangible and immediate implications for mitigating reactive N pollution and GHG emissions associated with the use of synthetic N fertilizers.

## Results

### Enhanced BNF and ammonium excretion in in-vitro conditions

**Fig. 2:**
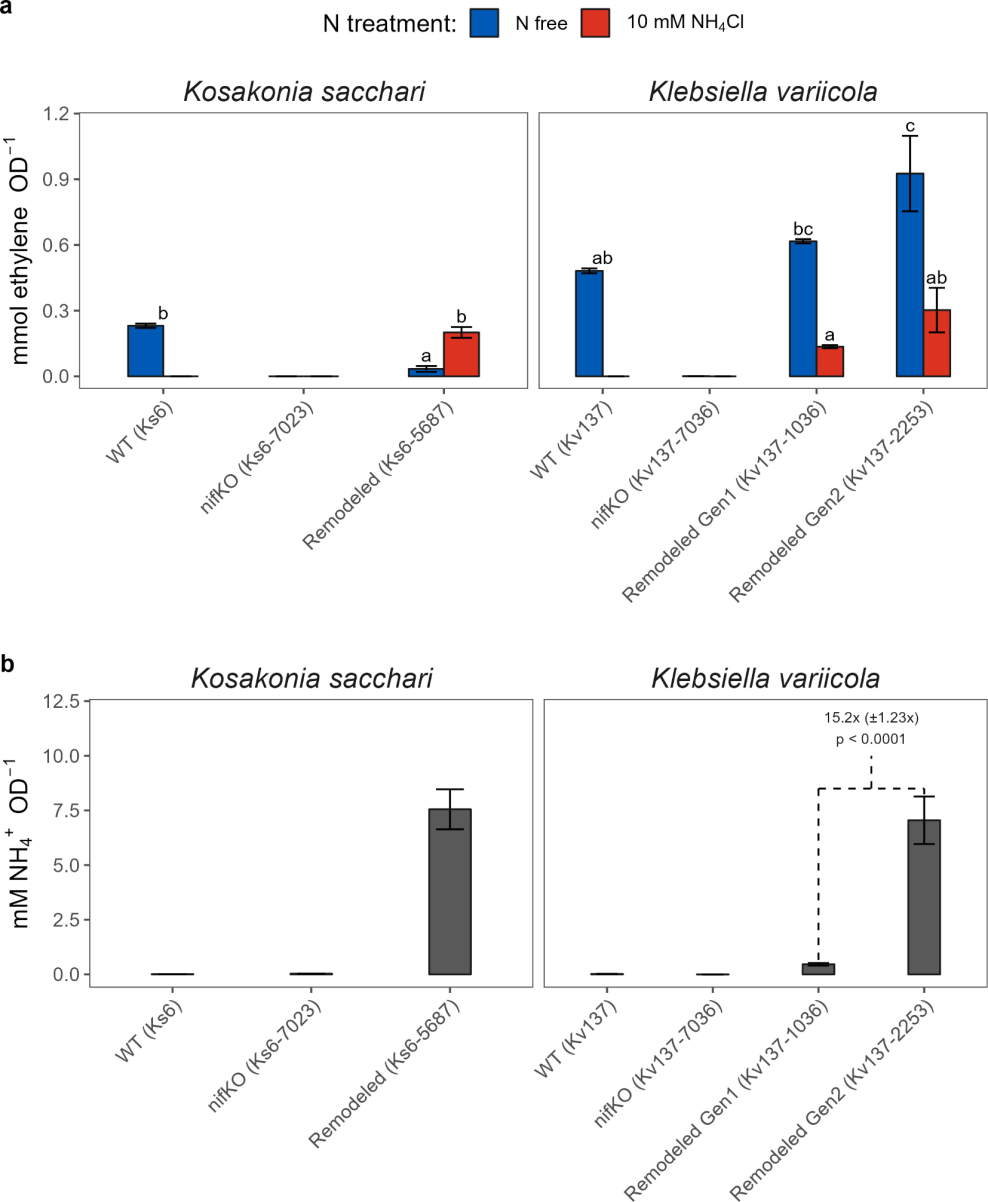
Evaluation of remodeled strains in vitro.** (a)** Biological N fixation activity was assayed using the Acetylene Reduction Assay (ARA) of individual strain growth cultures with wild-type (WT), *nif* gene knockout (*nif*-KO), and remodeled strains (supplemental Table S1) under N-free and N-rich conditions (10 mM NH_4_Cl). Bars represent sample means, and error bars show the standard error of four replicates. Letters denote the mean separation for the remodeled strain at the α = 0.05 level, using the Tukey adjustment. Wild-type strains (WT) with N-rich conditions and *nif-*KO strains were excluded from the test of hypothesis due to measurements often falling below the detection limit. **(b)** Ammonium excretion was assayed using the Megazyme Ammonia Assay (MAA) Kit (P/N K-AMIAR) in individual strain growth cultures. Bars represent the mean, and error bars show the standard error of eight replicates. Text annotation indicates the magnitude changes between *K. variicola generation* (Gen) 1 (*Kv*137-1036) and Gen 2 (*Kv*137-2253) edits as estimated by the test of hypothesis.

*In-vitro* acetylene reduction assay confirmed that *Ks*6-5687 and *Kv*137-2253 exhibit BNF activity under both N-free and N-rich conditions, whereas the wild-type *Kv*137 and *Ks*6 strains displayed BNF only in the absence of N (Fig. 2a). However, the edited *Kv*137-1036 and *Kv*137-2253 strains still showed some repression of nitrogenase in the presence of NH_4_Cl. To amplify NH_4_^+^ excretion of the previously engineered *Kv*137-1036 strain, we deleted the uridylyl transferase domain of GlnD in *Kv*137-2253, preventing this protein from activating the assimilation pathways (Fig. 1b-c and supplemental text S1). The edited strain *Kv*137-2253 excreted about 15 times more ammonium than the *Kv*137-1036 strain, underscoring the effectiveness of the *ΔglnD* edit over the previous *ΔnifL::PinfC* modification in *Kv*137-1036 (Fig. 2b).

### BNF and ^15^N_2_ incorporation in planta

**Fig. 3:**
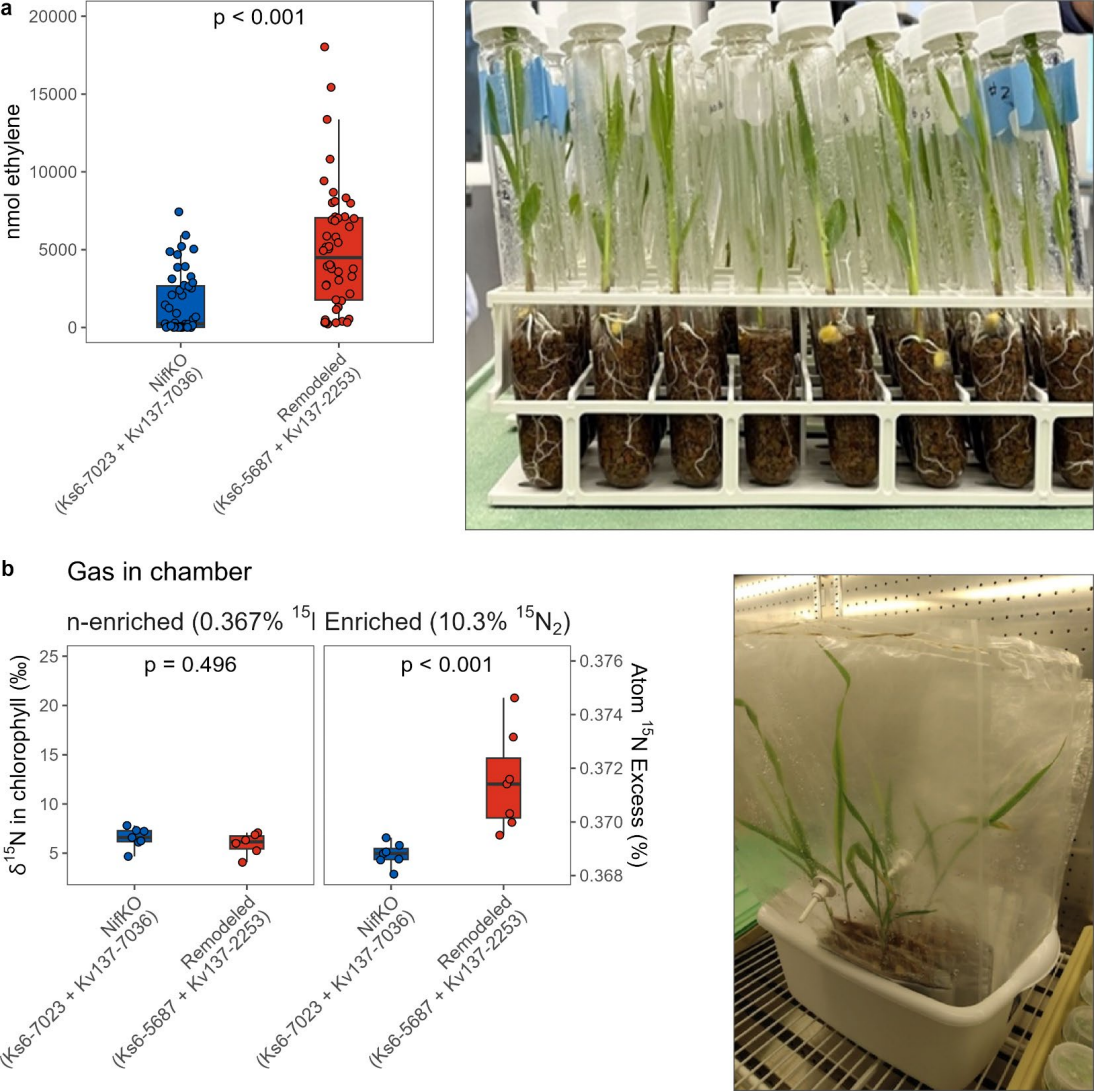
Evaluation of genetically remodeled strains in planta.** (a)** Measured biological N fixation activity of combined enhanced strains (*Ks*6-5687 + *Kv*137-2253) compared to combined *nif* gene knockout strains (*nif*-KO; *Ks*6-7023 + *Kv*137-7036) using the Acetylene Reduction Assay (ARA). Each point represents an individual plant replicate (n = 48 or 47). The p-value displayed corresponds to a two-tailed t-test and Welch’s correction for unequal variance**. (b)** Measured δ^15^N in chlorophyll extracted from 10-day-old plant shoots inoculated with enhanced strains and *nif*-KO combinations after incubating in sealed, 10 L bags supplemented with nonenriched (0.36% ^15^N_2_) or enriched gas (10% ^15^N_2_) for five days. Each box plot represents data from three experiments, comprising six or seven plants per treatment.

We have shown previously that both *Ks*6 and *Kv*137 strains have robust colonization and nitrogenase activity in the maize rhizosphere^26,27^. Inoculating agricultural fields with an assemblage of these two strains holds the potential to create a highly effective product capable of delivering more consistent performance across a broad spectrum of field conditions. Therefore, we measured the N-fixation ability of the remodeled strain assemblage (*Ks*6-5687 + *Kv*137-2253) when inoculated in maize seedlings grown in N-free hydroponic nutrient solution and gas-tight vials incubated with acetylene. Headspace gas analysis revealed increased acetylene reduction with the remodeled strains compared to plant tubes inoculated with the *nif-*KO strains (*p* < 0.001; Fig. 3a). This demonstrated the ability of these strains to fix N_2_ in a controlled plant environment. The small background ethylene signal observed in the *nif-*KO inoculated treatment could have originated from endogenous diazotroph nitrogenase activity given that this in-planta system was not completely sterile, or plant ethylene biosynthesis as a response to stress.

Labeling of N_2_ gas with ^15^N isotopes allows us to trace the movement and transformation of N, providing direct evidence of whether fixed N_2_ is incorporated into plant tissues. Results from ^15^N_2_ gas enrichment experiments conducted in a sterilized, N-free plant-growth pouch system (Fig. 3b) showed elevated atom% ^15^N concentration in the chlorophyll extract of plants inoculated with remodeled strains (*p* < 0.001), indicating the incorporation of atmospheric N_2_ into aboveground plant tissues. Conversely, no significant elevation in atom% with *nif-*KO strains was observed. Similarly, as expected, no significant increase in atom ^15^N for the enhanced or *nif*-KO treatments was observed when plants were incubated with non-enriched (0.367% ^15^N_2_) gas (Fig. 3b).

### BNF and plant response in small-plot field trials

#### ^15^N enrichment-dilution experiments

**Fig. 4:**
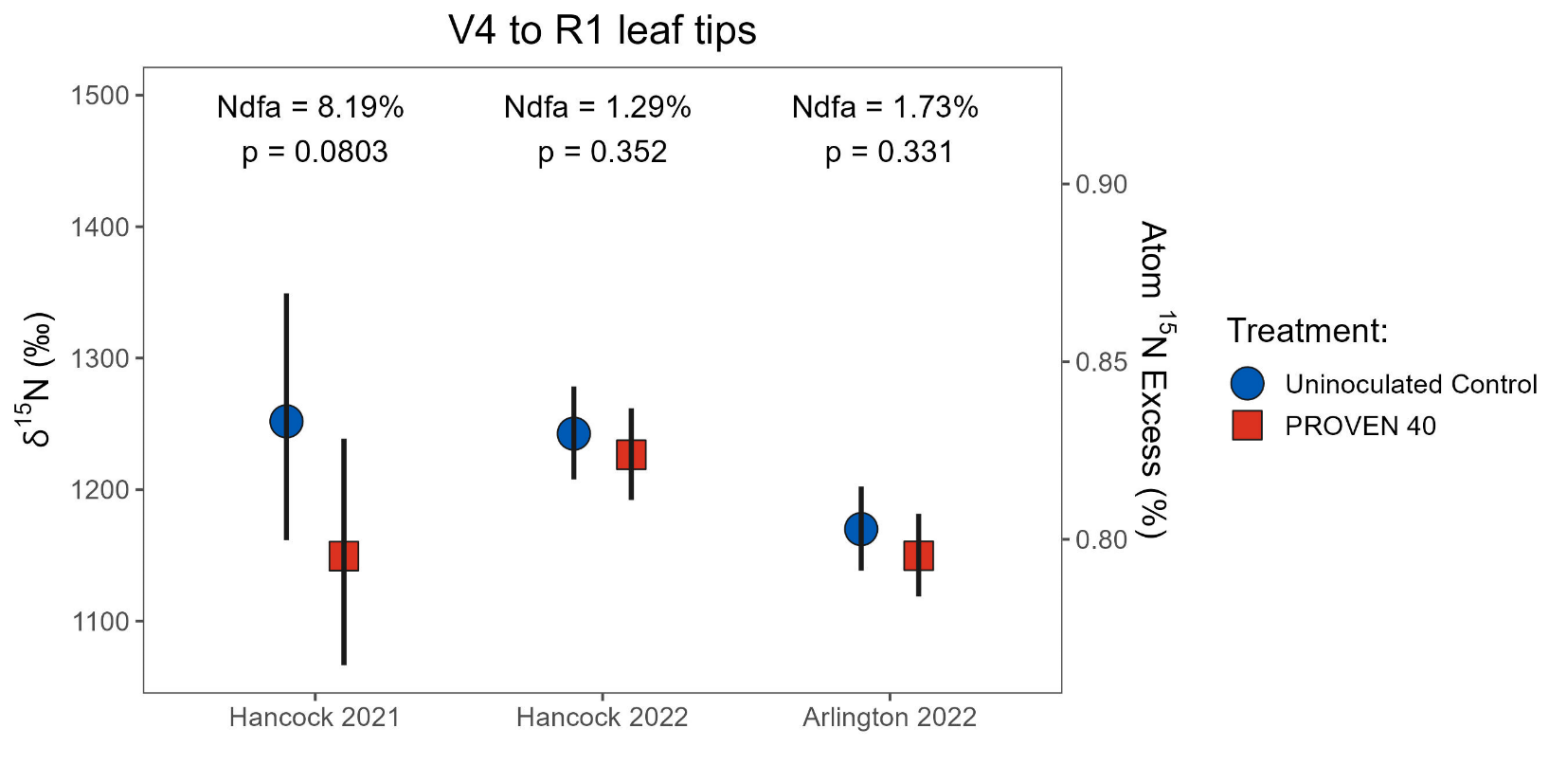
Estimated changes in leaf tip ^15^N abundance of field-grown maize using the ^15^N enrichment-dilution method. Leaf tips were collected from three experimental field trials in Wisconsin at five growth stages, from early vegetative to flowering. Symbols represent the estimated marginal means derived from fitted linear mixed effect models, back-transformed from the log scale. Error bars show 95% confidence limits around the means. Post-hoc pairwise comparisons of estimated marginal means between uninoculated control and PROVEN 40 were performed across growth stages, since their interactions by inoculation treatment were not significant (*p* > 0.1).

In small-plot field experiments conducted at the University of Wisconsin-Madison Hancock and Arlington Agricultural Research Stations, enriched ^15^N fertilizer was applied to soil and treated plots were inoculated with commercial-grade product containing both *Ks*6-5687 and *Kv*137-2253 strains (PROVEN 40; see supplemental Table S2 for experimental setup). Here, significant dilution δ^15^N compared to the uninoculated control was interpreted as a signal of crop N uptake of BNF. Samples collected from the 15 cm tip of the uppermost collared leaf (henceforth leaf tips) during across various growth stages showed marginally significant δ^15^N dilution in one out of three experiments but otherwise inconclusive results (Fig. 4). In the 2021 Hancock experiment, leaf tip dilution across all stages translated to 8.2% N derived from the atmosphere (Ndfa; *p* = 0.08). No significant dilution was detected in 2022 at either Hancock or Arlington. Because no significant interaction with the growth stage was detected in any of the experiments (*p* > 0.1), %Ndfa estimates across stages are reported for each experiment (Fig. 4).

#### Nitrogen fertilizer rate trials

**Fig. 5:**
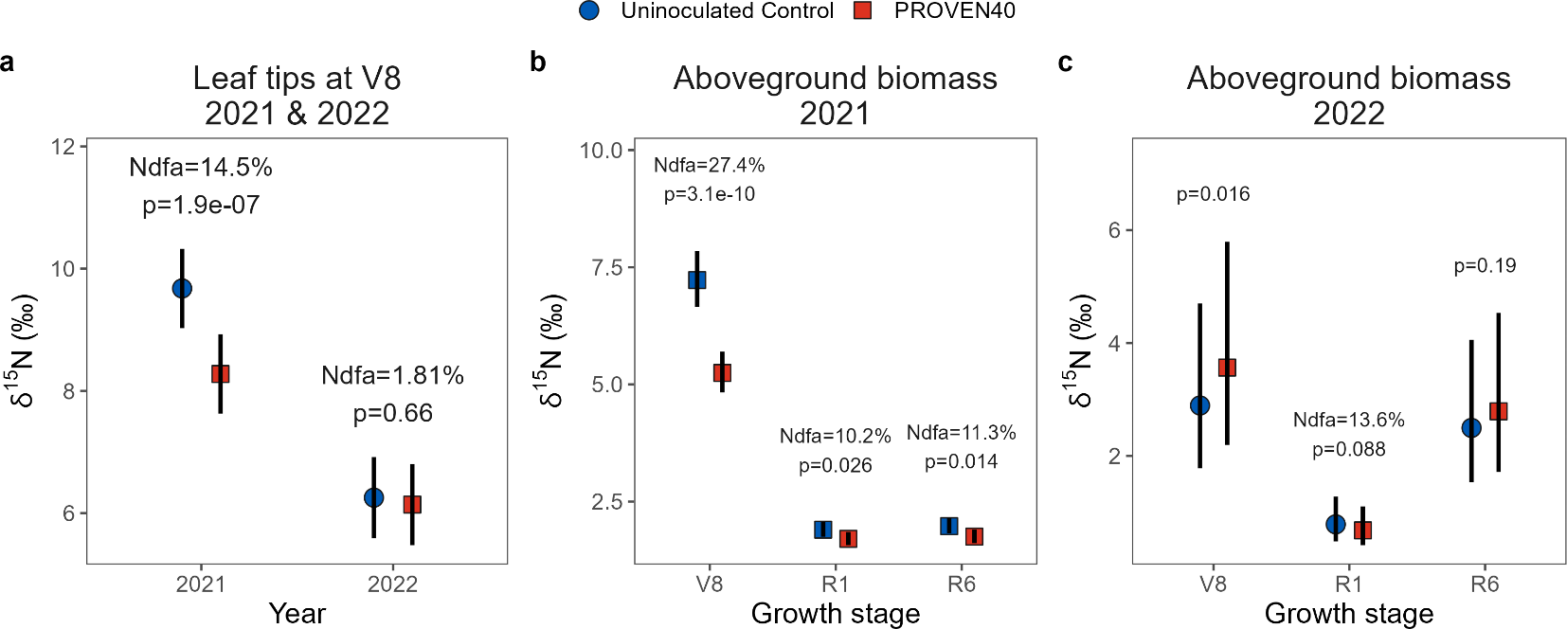
Estimated changes in δ^15^N natural abundance from a two-year N fertilizer rate trial at the Purdue University ACRE farm. **(a)** δ15N concentrations in leaf tips and above ground biomass in **(b)** 2021 and **(c)** 2022. Symbols represent the estimated marginal means derived from fitted linear mixed-effect models, back-transformed from the log scale. Error bars show 95% confidence limits. Percent N derived from the atmosphere (Ndfa) was calculated as the change from control δ^15^N abundance, divided by the marginal mean δ^15^N abundance of the control, assuming the “B” value of the equation is zero. Note that Ndfa values are only shown if a significant dilution in δ^15^N was observed in the inoculated control as assessed by a post-hoc pairwise comparison *t-test* with *p* < 0.10. At the ACRE farm, contrasts between treatments represent the mean effects across the six fertilizer rates, since fertilizer rate by inoculation treatment interactions were not significant (*p* > 0.1).

We now turn to results from a two-year N fertilizer rate experiment conducted at Purdue University Agronomy Center for Research and Education (ACRE) farm (supplemental Table S3 for experimental setup). Here we used the δ^15^N natural abundance approach, which relies on natural variations in ^15^N fractionation in atmospheric and soil pools (see Methods). We interpret the dilution of δ^15^N compared to the reference plots (i.e., uninoculated control with reduced N fertilizer rate) as a signal of BNF. Results revealed lower levels of δ^15^N in the leaf tips of V8 plants treated with PROVEN 40, compared to the uninoculated control in one year (14.5%; Ndfa *p* < 0.001) but not in the second year (Fig. 5a). No interaction with N fertilizer rate was observed. Additional measurements during the V8, flowering (R1), and physiological maturity (R6) growth stages showed substantial δ^15^N dilution in the above ground biomass in the first year (2021, Fig. 5b). The response was less pronounced in the second year (2022), with dilution in the aboveground biomass marginally detected only during the R1 stage (Fig. 5c). Note that 2022 at the ACRE farm was abnormally dry, particularly during the first 8 weeks post planting (supplemental Fig. S1). No interaction with the N fertilizer rate was detected in any of the years or growth stages.

**Fig. 6:**
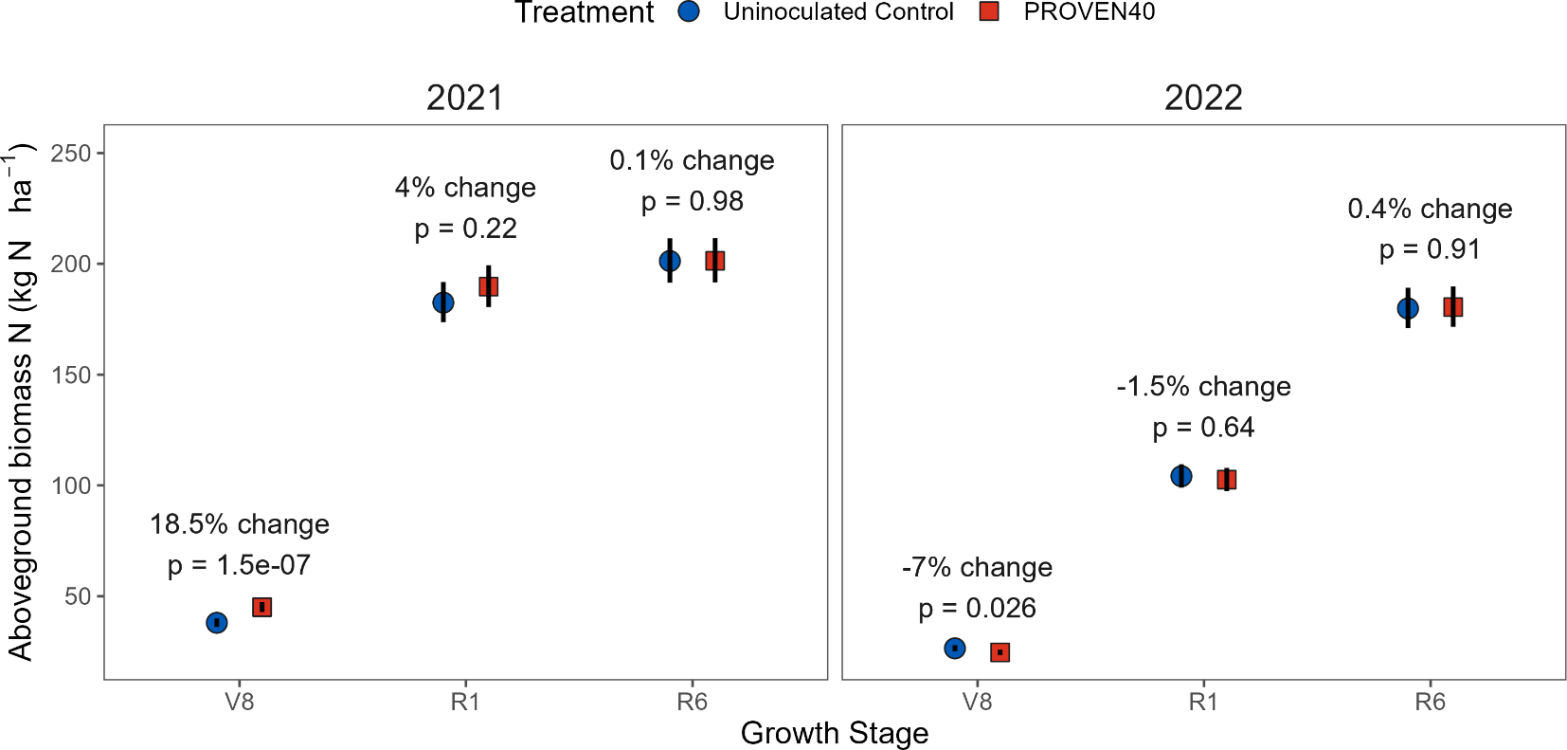
Estimated changes in aboveground biomass N from a two-year N fertilizer rate trial at the Purdue University ACRE farm. Symbols represent the estimated marginal means derived from fitted linear mixed-effect models, back-transformed from the log scale. Error bars show 95% confidence limits. Contrasts between treatments at each year and growth stage represent the mean effects across the six fertilizer rates, since fertilizer rate by inoculation treatment interactions were not significant (*p* > 0.1).

Similarly, a positive response in aboveground biomass N was only detected at V8 in 2021 (18.5% increase *p* < 0.001) but not at the R1 and R6 stages (Fig. 6). Based on the %Ndfa estimates for the R1 stage (Fig. 5b-c) and biomass N measurements, we estimate that PROVEN 40 provided 19.4 (95% CI: 3.25, 35.6) and 14.0 (95% CI: -0.95, 28.8) kg N ha^-1^ to the plant in 2021 and 2022, respectively (supplemental Table S4).

**Fig. 7:**
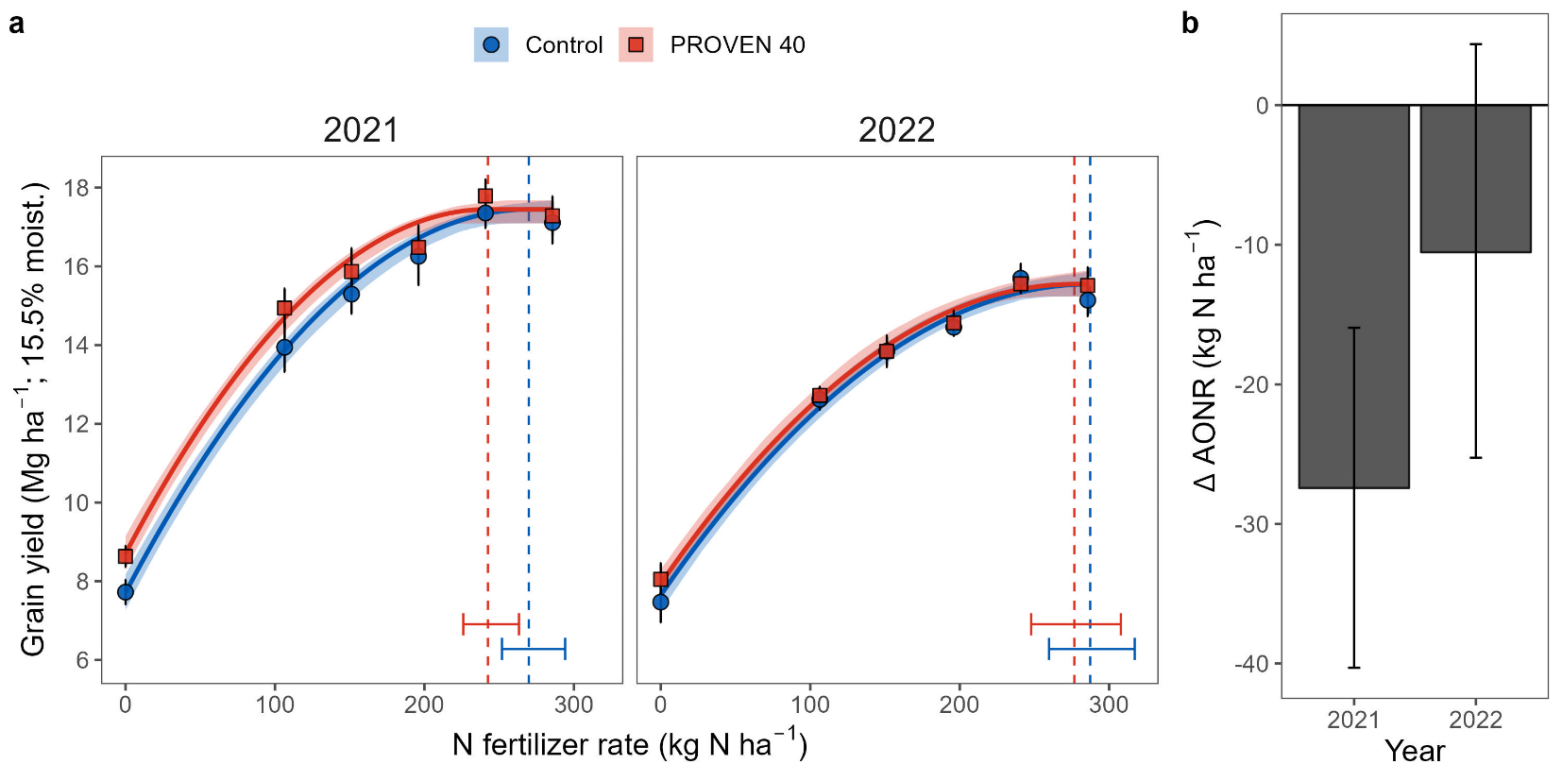
Effect of PROVEN 40 on the yield response to synthetic N fertilizer application rate and the agronomic optimum N fertilizer rate (AONR).** (a)** Estimated yield responses derived from experiments conducted in 2021 and 2022 at the ACRE farm. Symbols represent sample means, with their associated error bars denoting standard errors. Solid lines represent the best-fit curves using a mixed-effect nonlinear quadratic plateau model with replicate within a year as a random effect, with the surrounding shaded region representing the 95% confidence interval. Vertical dash lines intersecting the x-axis represent each respective AONR (i.e., the minimum N rate to achieve maximum yield), and error bars represent their estimated 95% confidence intervals. **(b)** Changes in the AONR relative to the uninoculated control. Error bars represent the 95% confidence intervals. All confidence intervals were estimated using residual-resampling bootstrapping (n = 300).

Employing a mixed-effect quadratic linear plateau modeling approach, we also examined differences in the maize yield response to the N fertilizer rate among inoculation treatments and site years as characterized by three curve parameters: yield with no N fertilizer, the slope of the response, and maximum attainable yield (i.e., when yield is no longer restricted by N; see supplemental information text S2 for more details on curve fitting). Specifically, we found that PROVEN 40 mainly affected the yield with no N fertilizer curve parameter, but the magnitude of the effect differed between the two site years (*p* = 0.064). The yield benefit of PROVEN 40 at zero N fertilizer was 0.7 Mg ha^-1^ less in 2022 than in 2021 (95% CI: -1.43, 0.024). Conversely, there was no evidence that PROVEN 40 affected the slope of the response (*p* = 0.995) or the maximum attainable yield (*p* = 0.67) across the two years (supplemental Tables S5 and S6). Utilizing the estimated parameters, we computed response curves across the synthetic N fertilizer rate continuum and determined the agronomic optimum N fertilizer rate (AONR; i.e., the minimum N rate to achieve the maximum attainable yield) at each combination of site-year and management treatment (Fig. 7a). This analysis revealed that PROVEN 40 reduced AONR by 27.4 kg N ha^-1^ (95% CI: -40.3, -15.9) in 2021 and by 10.5 kg N ha^-1^ (95% CI: -25.3, 4.37) in 2022 (Fig. 7b), compared to the uninoculated control. The AONR for the control was estimated at 270 kg N ha^-1^ (95% CI: 252, 294) and 287 (95% CI: 260, 308) in 2021 and 2022, respectively (Fig. 7a).

#### Inoculation rate trials

We examine data from 29 field trials (supplemental Table S7) conducted in 2022 in which maize seeds were inoculated prior to planting with PROVEN 40 with a ladder of inoculation rates (see Methods and supplemental Figure S2). At each site, inoculated plots received fertilizer at a reduced rate of 45 kg N ha^-1^ below the local business-as-usual (BAU) N fertilizer rate to elicit a small but measurable crop response to N (see supplemental Table S7 for details on N fertilizer management). Uninoculated plots with the reduced and BAU N fertilizer rates were also included to serve as controls.

**Fig. 8:**
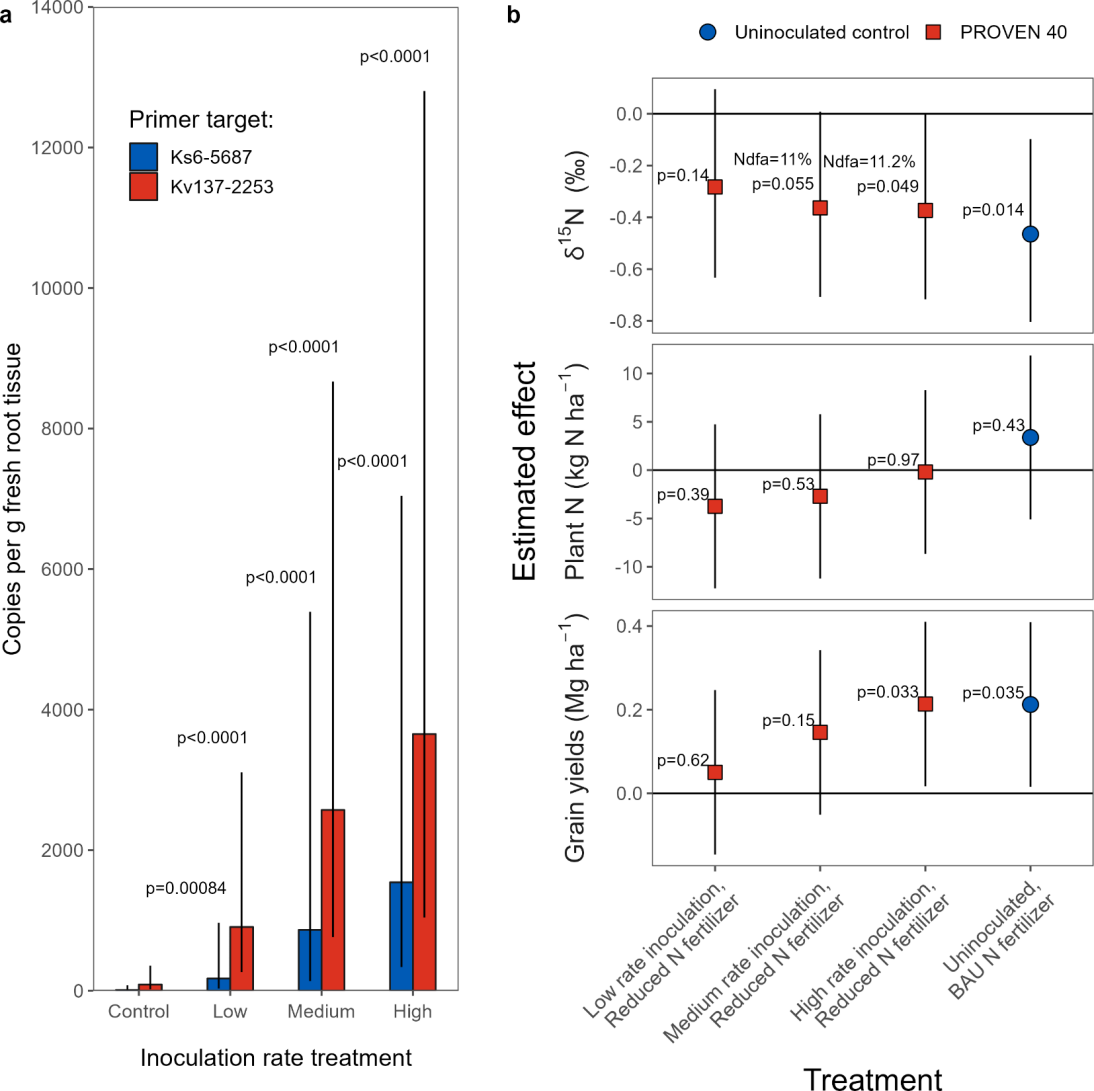
Estimates of root colonization, δ^15^N natural abundance and grain yield effects of treatments co-inoculated with Ks6-5687 + Kv137-2253 in small plot experiments.** (a)** Genomic DNA (gDNA) extracted from maize root samples collected during the V4-V5 growth stages at nine field sites. The inoculation treatments (Low, Medium and High) and control received 45 kg N ha^-1^ less than the business-as-usual (BAU) N fertilizer rate. Bars represent the estimated marginal means with the combined conditional and zero-inflated models, back-transformed from the *log(y* + *1*) scale. Error bars show 95% confidence interval of the means. P-values shown are for the contrast of each inoculated treatment versus their respective uninoculated control, adjusted using a Dunnett’s test approximation. (**b)** Estimated population-level effects of inoculation treatments and the BAU on δ^15^N abundance at VT-R1 (16 sites) and yields (28 sites). The control (uninoculated with reduced N fertilizer) was used as the baseline for all contrasts and had an estimated mean δ^15^N of 3.32 ‰ (95% CI: 2.73–3.99 ‰) and yield of 12.7 Mg ha^-1^ (95% CI: 11.8, 13.9). Percent N derived from the atmosphere (Ndfa) was calculated as the change from the negative control δ^15^N abundance, divided by the marginal mean δ^15^N abundance of the control, assuming the “B” value of the equation is zero. Error bars show 95% confidence intervals for the estimated effects.

Analysis of genomic DNA (gDNA) extracted from maize root tissue samples collected at a subset of sites (n = 9) and amplified via qPCR (see Methods) show significantly elevated levels of abundance of both *Ks*6-5687 and *Kv*137-2253 strains in the roots of V4-V5 maize plants compared to those in the uninoculated control (Fig. 8a), confirming that the remodeled strains can successfully colonize maize roots in fields. Minimal background amplification in the uninoculated control indicates that our primer sets were specific for our target strains in these field sites. Increasing inoculation rates show greater abundance in both remodeled strains (*p* < 0.001). We detected 130 and 37 times more gDNA copies in the high inoculation rate compared to the uninoculated control in *Ks*6-5687 and *Kv*137-2253, respectively. We also found evidence of differential abundance between the two strains, with populations averaging 4.8 times greater in *Kv*137-2253 compared to *Ks*6-5687 (*p* = 0.012).

Above ground biomass samples collected around flowering (VT-R1 growth stages) at a subset of trials (n = 16) and analyzed for δ^15^N natural abundance suggest significant δ^15^N dilution for the medium (mean = -0.36‰, 95% CI: -0.71, 0.0077) and high (mean = -0.37‰, 95% CI: -0.72, -0.0026) inoculation rate treatments (Fig. 8b). These translated to 11% Ndfa across both treatments (95% CI: 0, 22%). Note that, as expected, the dilution observed under the BAU (i.e., 45 kg of N ha^-1^ of additional N fertilizer over the reduced N) was also significant (mean = 0.46‰, 95% CI: -0.80, -0.098; Fig. 8b). Analysis of grain yields measured in 28 trials (Fig. 8b) show a significant yield increase compared to the negative control at the highest inoculation rate (0.21 Mg ha^-1^; 95% CI: 0.017, 0.41), but not for the medium and low inoculation rates. The BAU also exhibited a similar yield effect (0.21 Mg ha^-1^; 95% CI: 0.016, 0.41). No significant effects on aboveground biomass N at flowering (VT-R1) were detected for any of the treatments (*p* > 0.1; Fig. 8b). Based on these measurements, we estimate that PROVEN 40 provided on average 21.2 (95% CI: -0.16, 42.6) kg N ha^-1^ to the plant by VT-R1 (supplemental Table S8).

### Inoculation response in commercial-scale fields

#### Changes in crop N status

**Fig. 9:**
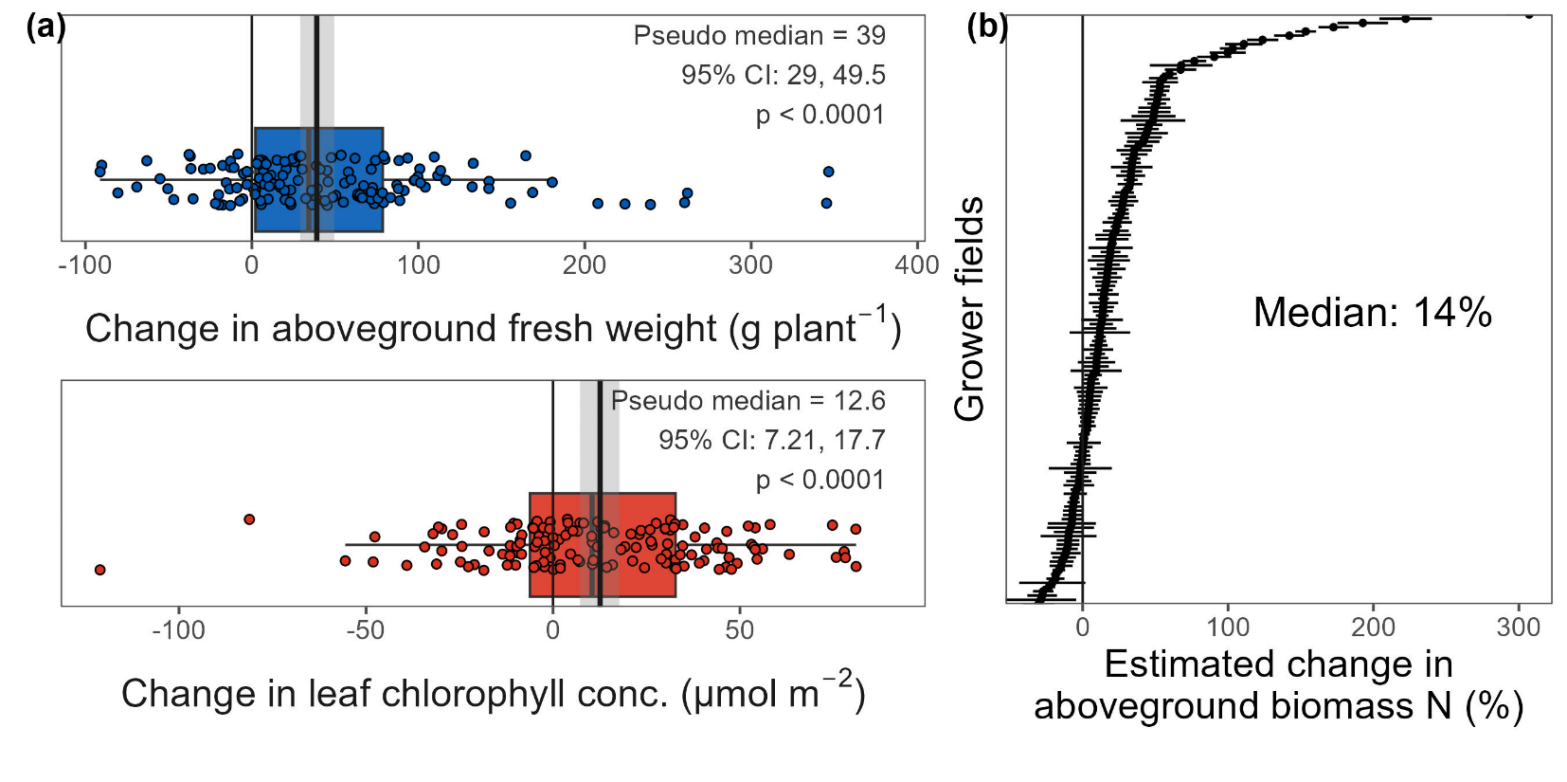
Early-season plant response to N supplied by remodeled strains.** (a)** Measured changes in aboveground fresh weight and leaf chlorophyll concentrations between plant samples that received the local business-as-usual (BAU) N fertilizer rate and samples inoculated with PROVEN 40 with 39 to 45 kg N ha^-1^ less than the BAU. Samples were collected during vegetative growth (V6 to VT) at 135 on-farm side-by-side grower trials (supplemental Fig. S3). Points indicate the average treatment difference at each site. The hypothesis was tested using a non-parametric Wilcoxon paired t-test with continuity correction. **(b)** Estimated changes in aboveground biomass N at the sites between PROVEN 40 and BAU treatments. Sites are shown ranked from low to high. Points show the predicted change at each site, and error bars show 95% confidence intervals. Estimates are extrapolated using the multiple linear regression model of aboveground fresh weight and leaf chlorophyll concentration measurements to aboveground biomass N at a subset of nine sites (supplemental Fig. S4).

We analyzed maize aboveground fresh biomass and leaf chlorophyll concentration in samples collected from the sixth leaf stage (V6) up to tasseling (VT) to explore maize plant response to inoculation with PROVEN 40 on real-world conditions. Our study encompassed 135 commercial-scale grower fields during 2022 and 2023 (supplemental Fig. S3), comparing uninoculated field zones with BAU N fertilizer rate against inoculated zones that received 39 to 45 kg N ha^-1^ less than the BAU. Analysis indicates that inoculation with PROVEN 40 led to positive changes in plant fresh weight (39 g plant^-1^; *p* < 0.0001), as well as in leaf chlorophyll concentrations (12.6 µmol m^-2^; *p* < 0.0001; Fig. 9a). We estimate a median increase in aboveground biomass N of 14% across all sites (Fig. 9b), based on a regression model of data from a subset of nine sites where aboveground biomass N was also measured (cross-validated R^2^ = 0.89; see methods and supplemental Fig. S4).

#### Yield response to practice change

Finally, we examine yield impacts of practice change using a dataset of 58 commercial-scale fields in 2021. Here, each field also included zones inoculated with PROVEN 40 and received 39 to 45 kg N ha^-1^ less N fertilizer than the BAU, and BAU control zones (supplemental Fig. S5). Field-level mean yield comparisons between BAU and PROVEN 40 with reduced N fertilizer plots showed no significant reduction in yield across all sites (*p* = 0.87, Table 1). The minimum detectable effect of the test is 0.166 Mg ha^-1^ or 1.2% of the population mean yield estimate, underscoring the robustness of this finding. Notably, field areas inoculated with PROVEN 40 at the reduced N fertilizer exhibited an 8% decrease in the coefficient of variation (CV) of yield compared to uninoculated BAU counterparts (*p* = 0.013, Table 1).

**Table 1:** Comparison of maize mean yield and within-field yield variability between two N fertility management systems.

Metric	Treatment^a^	Estimated Marginal Mean (± SE)	Contrast Type	Effect size (± SE)	DF	P( >|t|)	MDE ^d^
Yield Mean (Mg ha^-1^)	BAU	13.43 ± 0.306	PROVEN 40_ReducedN_ – BAU	-0.0135 ± 0.083	57	0.871	0.166
	PROVEN 40_ReducedN_	13.42 ± 0.306					
Yield CV^b^ (%)	BAU	13.61 ± 0.755^c^	PROVEN 40_ReducedN_ / BAU	0.921 ± 0.03	57	0.0129	0.0591
	PROVEN 40_ReducedN_	12.53 ± 0.695^c^					

^a^ Uninoculated maize received the business-as-usual (BAU) N fertilizer application rate. In contrast, maize inoculated with PROVEN 40 received uniform reductions of 39–45 kg N ha^-1^ relative to the BAU at the respective location in 58 large side-by-side trials (approximately 11.26 ha per treatment area) located across the primary maize-growing region in the U.S. (supplemental Fig. S3).

^b^ Coefficient of variation: (within-field standard deviation / within-field mean) × 100.

^c^ Back-transformed from the log scale.

^d^ MDE: Minimum detectable effect with α = 0.05.

## Discussion

The evidence presented here broadly supports the hypothesized mechanism of enhanced BNF by the remodeled strains, representing a source of N typically not available to maize grown with high levels of synthetic N fertilizer. Our research confirms that the genetic remodeling strategy for *Ks*6-5687 and *Kv*137-2253 (Fig. 1) effectively derepresses BNF activity in N-rich systems and enhances ammonium excretion by orders of magnitude above the respective wildtype strains, displaying a 15-fold increase relative to our previously edited *Kv*137-1036 strain^27^ (Fig. 2b). In-planta BNF is demonstrated by the transfer of ^15^N-enriched N_2_ gas from the rhizosphere to the chlorophyll of maize in controlled plant environments (Fig. 3b). This was partially corroborated by the ^15^N dilution detected in a portion of the field experiments (Figs. 5 and 7b). We also show that maize N status during mid vegetative growth was positively affected by inoculations in small-plot experiments (Fig. 6) and grower fields (Fig. 9), although differences in aboveground biomass N could not be detected during the VT-R1 and R6 growth stages (Figs. 6 and 8b). Nonetheless, we observed similar yield responses of the BAU fertilizer rate and PROVEN 40 under a fertilizer reduction of up to 45 kg N ha^-1^ in both small-plot experiments (Fig. 8b) and on-farm trials (Table 1). The latter suggests that the gene-edited strains have the potential to mitigate the risk of yield penalties when synthetic N fertilizer inputs are reduced, although the environmental conditions that both crop and microbes experience likely impact the fertilizer replacement potential.

### Field BNF estimates and uncertainties

Evidence from the field studies suggest that the *Ks*6-5687 and *Kv*137-2253 strains can successfully colonize maize roots, persist to at least the V4-V5 stage (Fig. 8a) and retain the potential to provide BNF to maize in N-rich field environments. These remodeled strains are estimated to contribute 11% Ndfa in the above ground biomass by the VT-R1 crop growth stage, which translates to 21.2 kg N ha^-1^ (supplemental Table S8). This %Ndfa estimate is comparable to the R1 %Ndfa (14–19 kg N ha^-1^; supplemental Table S4) and the estimated reduction in AONR (10.5–27.4 kg N ha^-1^; Fig. 7b) observed at the ACRE farm. These are also within the range of rates previously achieved with wildtype strain inoculation in cereal crops^23^, which can contribute 13–26 kg N ha^-1^ in maize, 3–40 kg N ha^-1^ in wheat, and 18–51 kg N ha^-1^in rice^31^. However, these previous estimates originate from experiments in soils receiving little or no N fertilizer, except for one study in rice^24^ which reported a > 80% reduction in BNF activity in soils fertilized with 125 kg N ha^-1^ or more. Therefore, our results represent one of the few datasets suggesting heightened BNF activity in N-rich soils, which we accomplished through our nitrogenase derepression and ammonium excretion gene edits.

The large uncertainty in Ndfa estimates, however, remains a major barrier in our analysis. Note that population-level estimate of Ndfa across 16 sites in the inoculation rate trials was found to be within -0.16 and 42.6 kg N ha^-1^with a 95% confidence (supplemental Table S8). Another study^28^ reported an even wider range of Ndfa in indigenous maize landraces (29–82%; 4–122 kg N ha^-1^). Rates of free-living and associative BNF are known to be highly variable, due to spatial–temporal gradients in its underlying drivers such as temperature, moisture, pH, oxygen concentration and carbon substrate availability^29^. Indeed, the N fertilizer rate study at the ACRE farm found limited evidence of BNF in 2022 (Figs. 5 and 6), a year in which the site received only 77 mm of rainfall within the first 50 days post-inoculation (~ 36% of the climatic mean; supplemental Fig. S1). Dry soil conditions are known to have adverse effects on the soil microbiome, both directly and indirectly due to changes in the composition and flux of carbohydrates from roots^30–32^. We expect the effects to be more pronounced in diazotrophs given the potential for oxygen damage to the nitrogenase enzyme, as dry soil conditions allow for increased oxygen diffusion into the rhizosphere^33^. However, we lack data to determine the extent to which survival and BNF by the remodeled strains is modulated by drought.

Leaf tip measurements from the ^15^N enrichment-dilution experiments, although directionally consistent with our other results, were much less conclusive (Fig. 4). Yet, the generalizability of %Ndfa estimates from leaf tips to the entire plant remains unclear. While a previous study found that %Ndfa in the third-youngest leaf was representative of the whole plant (including the roots) during the second to sixth month post-planting^28^, these findings were on indigenous Mexican landraces of maize grown in N-depleted soils. In contrast, our trials evaluated commercial hybrids (supplemental Tables S2, S3, and S7) in well fertilized conditions, and tissue collected was from the youngest collared leaf. A key assumption is that these leaf tip samples reflect a recent and transient flux from BNF rather than a whole-plant estimate. To the best of our knowledge, however, this hypothesis has not been validated, and remains a focal point for future research.

Free-living and associative diazotrophs, *including K. variicola*, are also known to offer plant health benefits beyond BNF, including mineral solubilization, phytohormone production and stress tolerance^34,35^. It is then possible that additional mechanisms could also be influencing our Ndfa estimates. In all our field ^15^N tracer studies, we assume that the inoculated crop and its respective reference crop (i.e., the uninoculated control) have similar rooting characteristics and N assimilation dynamics from fertilizer and soil (apart from BNF from the inoculum). Thus, we attribute the relative decrease in ^15^N in the inoculated crop to the proportion of plant N uptake from root-associated BNF carried out by *Ks*6-5687 and *Kv*137-2253. However, since synthetic N fertilizers typically exhibit lower δ^15^N abundance than soil^36,37^, the observed decrease in δ^15^N could partly stem from greater fertilizer recovery efficiency if larger root biomass or changes in root architecture are occurring in response to other growth promoting mechanisms. Although this scenario could mean that Ndfa is being overestimated, greater fertilizer recovery would nonetheless have positive implications for mitigating NO_3_ leaching and N_2_O emissions. Further research therefore is needed to disentangle these complex processes. Given that Ndfa methods (e.g., ^15^N enrichment-dilution, ^15^N natural abundance, N balance) are all susceptible to large uncertainty, especially in cereal crops, here we use multiple methods as previously suggested^38^. We refer to *ref.*^23^ for additional review of the methodological challenges of measuring BNF in cereal crops.

### Impacts of BNF on maize N status and growth

Results also demonstrate a potential positive influence of PROVEN 40 inoculation on the early-season N status of maize plants in small-plot trials (Fig. 6) and on-farm trials (Fig. 9). In the latter, we construe that the enhancements in fresh biomass and leaf chlorophyll content in inoculated plants suggest heightened rates of N assimilation during the initial growth stages, given that these parameters are known to be correlated to crop N status^39–42^. This correlation was corroborated by ground truth, aboveground biomass N measurements in a subset of sites (supplemental Fig. S4). That these improvements are driven by inoculation is particularly convincing given the concurrent 39–45 kg N ha^-1^fertilizer rates reduction in inoculated plants. This early-season advantage could occur because BNF is more spatially accessible to plant uptake than soil N during the early season. Not only are maize root systems small at this point, but microbially driven soil N mineralization is typically limited by cool temperatures, particularly in poorly drained soils with no-till or thick residue cover because these conditions maintain lower soil temperatures longer in the spring^43^.

This N uptake advantage appears to largely disappear by VT-R1 (Figs. 6 and 8b), perhaps because N mineralization or other sources of soil N can typically fill the gap. Nevertheless, the increase in early-season N uptake resulting from BNF could still have positive impacts on yield potential. Pre-V8 N deficiency can cause irreversible reductions in ear size and kernel number^44^, primarily due to its detrimental impact on floral primordia differentiation around V5-V6^45^. Although compensatory mechanisms can increase individual kernel weights^44,46^, reduced kernel count generally leads to lower yields. In this context, BNF could help maximize kernel number potential by decreasing early-season N stress. However, further research is needed to test this hypothesis. Moreover, the final effect on grain yields may depend on the inoculum’s ability to sustain a continued supply of fixed N beyond the early-season and on sufficient soil N supply required to meet the augmented N supply driven by increased yield potential. There is little evidence supporting BNF activity after flowering, given the lack of difference in aboveground biomass N at VT-R1 (Figs. 6 and 8b), and Ndfa between R1 and R6 at the ACRE farm (Fig. 5b-c). Although there is evidence of strains persisting into the V4-V5 stages (Fig. 8a), fitness costs associated with the nitrogenase derepression and ammonium excretion gene edits^26^could hinder their long-term competitiveness in the soil microbiome, such that declining populations later in the growing season would be expected. If the above is true, then inoculation might be most effective in environments where N uptake occurs mainly during vegetative growth, similar to what has been shown for early in-season N fertilizer application^47^.

### Implications

Results from Table 1 could suggest that, on the aggregate, on-farm reductions of N fertilizer inputs of 39–45 kg N ha^-1^ with PROVEN 40 inoculation exhibit no deleterious yield effects compared to the BAU. Note that the power of the test on Table 1should allow us to detect any difference greater than 0.17 Mg ha (or 1.2% of the population mean). This evidence, however, should be interpreted with caution given that it is likely that some of the BAU fertilizer rates exceeded their site-year AONR, in which case a lack of response is to be expected. Indeed, grower-determined fertilizer rates are based on a combination of site-specific knowledge and risk tolerance^19^ and on average exceed optimum rates by 17 to 28 kg N ha^-1^^48^ . However, other data presented here may also support this conclusion, including: 1) the comparable yield results from the reduced N fertilizer high inoculation rate treatment and the BAU (Fig. 8b); 2) decreases in AONR in 2021 at the ACRE farm (Fig. 7); and 3) the reduction in within-field yield variability with inoculation (Table 1).

Although a 39–45 kg N ha^-1^ reduction in N fertilizer rate represents a modest fraction of the total amount of N fertilizer applied to maize, widespread implementation could significantly reduce fertilizer consumption. The *Ks*6-5687 + *Kv*137-2253 strain assemblage has been commercialized as PIVOT BIO PROVEN® 40 in the U.S^49^ with an estimated 2 million ha of adoption area in 2023. In many cases, inoculation has been accompanied by reductions in N fertilizer rates, which avoids GHG emissions from fertilizer manufacturing (⪝ 2.6 kg CO_2_equivalents per kg of N applied)^50^, and has potential to avoid soil N_2_O emissions and NO_3_ leaching. However, further research is necessary to confirm and quantify this impact. Nevertheless, the prospect of reducing N fertilizer requirements in maize through BNF represents an incremental step towards decoupling U.S. cereal crop production from synthetic N fertilizer use. However, as discussed above, environmental constraints could be important in modulating product efficacy. Therefore, a comprehensive assessment of the role of management and environmental conditions in its effectiveness and impacts on N_2_O emissions and NO_3_ leaching are the crucial next steps for developing a more mechanistic understanding of how remodeled strains affect the cropping system. This new knowledge will enable us to optimize efficacy and evaluate the complete life cycle impacts of the technology, getting us closer to the goal of replacing synthetic N fertilizers.

## Methods

### Laboratory assays

#### Genetic modifications and strain remodeling

*Klebsiella variicola* (*Kv*137) and *Kosakonia sacchari* (*Ks*6) parent strains, previously identified as robust maize root colonizers^26,27^, were engineered through non-transgenic methods to enhance their BNF activity under N-rich conditions, resulting in modified strains Kv137-2253 and Ks6-5687. The engineering involved editing the *nifLA* operon to boost nitrogenase protein activity and editing the *glnD* and *glnE* genes to facilitate the extracellular release of NH_4_ (Fig. 1b-c). The rationale and details behind the gene edits are provided in the supplemental information (supplemental text S1).

#### In-vitro assays

##### Acetylene reduction assay

A modified version of the acetylene reduction assay (ARA) described by *ref.*^27^ was used to measure nitrogenase activity in pure culture conditions. Strains were cultured from single colonies into 5 mL of super-optimized broth (SOB) for 24 h (30 °C, aerobic). The growth culture (1 mL) was then added to 25 mL of minimal media supplemented with 10 mM glutamine and grown for 24 h (30 °C, aerobic). The growth culture (0.6 mL) was then added to 2.4 mL of minimal media or 2.4 mL of minimal media supplemented with 10 mM NH_4_Cl in airtight culture tubes prepared in an anaerobic chamber and grown for 4 h (30 °C, anaerobic). A headspace of 10% was replaced by an equal volume of acetylene, and incubation continued for an additional hour. A gas-tight syringe was used to remove 2 mL of headspace in preparation for ethylene production quantification using an Agilent 7890B gas chromatograph equipped with a flame ionization detector. The initial culture biomass was compared to the end biomass by measuring optical density at 590 nm (OD_590_). The change in biomass and ethylene concentration was used to calculate mmol ethylene OD^-1^, a proxy for BNF. To establish a negative control, we knocked out the central nitrogenase subunit *nifH*, deleting the entirety of the *nifH* gene in the background of both remodeled strains (Ks6-7023 and Kv137-7036). The absence of BNF in the *nifH* gene knockout (*nif-*KO) strains was confirmed via ARA prior to use as a negative control. BNF in strains of the same wildtype lineage was compared using a one-way ANOVA and post-hoc Tukey test across four replicate experiments. Treatments with a substantial number of measurements below the detection limit of the assay were excluded from the analysis.

##### Ammonium excretion assay

The ammonium excretion assay measures the excretion of fixed N in the form of ammonium using batch cultures in DeepWell plates. Strains were propagated from a single colony in 1 mL well^-1^ SOB in a 96-well DeepWell plate. The plate was incubated for 24 h (30 °C, 200 rpm) and then diluted 1:24 into a fresh plate containing 1.15 mL well^-1^ of minimal media containing 10 mM glutamine. Cells were incubated for 24 h (30 °C, 200 rpm) and then diluted 1:10 into a fresh plate containing minimal medium without glutamine. The plate was transferred to an anaerobic chamber (Coy Laboratory Products, Inc. Grass Lake, MI, U.S.) with a gas mixture of > 98.5% N, 1.2–1.5% hydrogen, and < 30 ppm oxygen and incubated at 1320 rpm at room temperature for 72 h. The initial culture biomass was compared to the end biomass by measuring OD_590_. Cells were then separated by centrifugation. The supernatant from the reactor broth was assayed for free ammonium using the Megazyme Ammonia Assay Kit (NEOGEN Corp., Lansing, MI, U.S.) and normalized to biomass at each time point to calculate mmol NH_4_^+^ OD^-1^. The experiment was replicated eight times. After removing measurements below the assay’s detection limit, the ammonium excretion of remodeled strains of the same wildtype lineage was compared using a two-tailed t-test with log-transformed measurements to correct for heteroskedasticity between treatments.

#### In-planta assays

##### Maize seed sterilization and germination

Maize seeds used in the experiments correspond to commercially available maize hybrids sourced from local seed dealers, unless stated otherwise. Seeds were sterilized before use in experiments to reduce the effects of environmental microorganisms. Maize seeds were surface sterilized with 70% isopropanol for two minutes and 2.5% bleach for 15 min. Seeds were rinsed thrice in sterile water and then immersed in a fungicide solution (Captan at 60 mg mL^-1^) overnight with gentle shaking. After three rinses with water, seeds were germinated on Petri dishes in the dark for three to four days on wet paper towels.

##### Acetylene reduction assay

This experiment aimed to investigate the N-fixing capabilities of the engineered bacterial strains and their impact on ethylene production in maize plants. For this, maize seeds were sterilized and germinated before planting in 70 mL individual tubes with Turface® (calcined, non-swelling illite clay) as the growth medium and N-free Hoagland’s hydroponic nutrient solution for plant nutrition. The plants were grown uncapped for four days in a growth chamber (16 h, 28 °C days and 8 h, 25 °C nights). Bacterial cell suspensions were grown from frozen glycerol stocks and normalized to OD_600_ = 0.8. The cell suspensions were combined in equal volume into two groups: 1) enhanced strains and 2) *nif-*KO strains, both in enhanced strain backgrounds. The combined bacterial suspensions were mixed 1:2 with semisolid Fahräeus medium and used to inoculate the four-day seedlings (8 mL plant^-1^). The tubes were sealed, and acetylene gas was introduced into the headspace. After six days, 2 mL of headspace samples were collected to measure ethylene production. After testing for normality with a Shapiro-Wilks test, data were analyzed with a two-tailed t-test with Welch’s correction for unequal variance. We excluded one tube in the *nif-*KO treatment from the analysis because of unexpectedly low amounts of acetylene gas, indicating a possible leak in the tube.

##### ^15^N gas enrichment assay

This experimental setup aimed to directly measure the extent to which growing maize plants incorporate N fixed by the engineered strains. Enhanced strains (Ks6-5687 and Kv137-2253) and their *nif-*KO counterparts (Ks6-7023 and Kv137-7036) were cultured on TY solid medium at 30 °C for 12 h. Maize hybrid PHJ89 x PH207^51,52^ seeds were provided by the University of Wisconsin Corn Silage and Biofeedstock Breeding Program. Seeds were sterilized and germinated before plantlets were transferred into CYG seed germination pouches, each containing three plantlets and sterile water. These pouches were placed in a plant growth chamber with controlled lighting and humidity. The five-day-old plantlets were inoculated with 10 mL of each bacterial strain and grown individually in tall plastic containers for seven days before being transferred into gas sampling bags.

For isotopic labeling, ^15^N gas (^15^N_2_) or industrial-grade N gas (N_2_) was injected into the gas bags to create control and ^15^N enriched conditions at the same oxygen level. To ensure accurate ratios of ^15^N:^14^N, the GB6000 gas mixer (MCQ Instruments, Rome, Italy) was utilized, resulting in 10.33% ^15^N in the ^15^N-enriched bags and 0.3663% in the control. Shoots and roots of the inoculated plants were harvested separately after five days in the growth chamber and prepared for analysis. Chlorophyll was extracted from two to three shoots following the methodology in *ref.*^53^ and sent to the isotope ratio mass spectrometry facility for ^15^N analysis. ^15^N incorporation between plants inoculated with *nif-*KO and remodeled strains was compared using two-tailed student t-tests. Prior to analysis of variance, Shapiro–Wilk and Levene tests were used to establish normality and equal variance, respectively.

### Field research trials

#### Product fermentation and formulation

All field-applied microbial inoculants were produced using standard proprietary fermentation methods and media selected to promote microbial growth and longevity. The fermentation broth was diluted using proprietary formulations (these included but were not limited to disaccharides, sugar polymers, buffers, and salts) to achieve a minimum titer (1 × 10^8^ CFU mL^-1^) in the formulated product. Inoculants were distributed to growers in bladders containing the ready-to-use liquid formulation to be applied in-furrow during planting. In the cases were inoculated seed was used, the fermentation broth was concentrated (> 10x) and freeze-dried (-30⁰C) to a powder. Finally, the powder was resuspended in a liquid formulation and applied to seeds prior to planting. Growers were instructed to store products away from direct sunlight and refrigerated or at room temperature to ensure product viability for the duration of its shelf life.

#### University of Wisconsin-Madison experiments

Three experimental field trials were conducted across two growing seasons in 2021 and 2022 at the University of Wisconsin-Madison's Hancock and Arlington Agricultural Research Stations to assess the transfer of biologically fixed N from modified bacterial strains to maize plants using the ^15^N enrichment-dilution method. The experiments featured three-row plots with maize hybrid PHJ89 x PH207, following a randomized complete block (RCB) design with 12 replications in 2021 and 30 in 2022. To enrich the field with ^15^N, a 1.0 atom % ^15^N solution was applied using ammonium sulfate one day before planting. Aiming for uniform distribution of the isotope across the field, ^15^N fertilizer was mixed with starter fertilizer, evenly distributed during planting. Throughout the growing season, side dress fertilizer applications were made per the farm's recommendations, accompanied by a 1.0 atom % ^15^N enrichment in either powder or liquid form, ensuring uniform incorporation into each row of the experiment. The process of inoculation with Kv137-2253 + Ks6-5687 involved labeling maize seeds with specific treatments, soaking the seeds before planting, and subsequently applying a second inoculation when plants reached the second and third leaf stages (V2-V3). Special care was taken to avoid contamination using dedicated equipment for each treatment. Leaf tip samples were systematically collected, dried, ground, and sent to the Cornell Stable Isotope Laboratory (Ithaca, NY, U.S.) for stable isotope analysis. Reported values are expressed as δ^15^N and atom % ^15^N excess, assuming atmospheric N_2_ gas is 0.37% ^15^N.

#### Purdue University experiments

Field experiments were conducted at Purdue University's Agronomy Center for Research and Education (ACRE) during the 2021 and 2022 growing seasons. These experiments featured six-row plots planted with maize hybrid P1359AM using a RCB split-plot design with eight replications. The main plots comprised six N fertilizer treatments ranging from 0 to 286 kg N ha^-1^. At the same time, the subplots included the inoculation treatment with commercial-grade inoculant with *Kv*137-2253 and *Ks*6-5687 strains (PROVEN 40) and a uninoculated control (NTC). In both years, the experimental fields were planted with soybeans in the previous year, and were tilled in the previous fall before planting. Biomass samples were collected at V8, flowering (R1), and maturity (R6) from 10 consecutive plants per plot. At V8, tips (15 cm) from the uppermost collared leaf were collected from 20 representative plants per plot. Samples were dried at 60 °C for five days, weighed ground to 1 mm, and analyzed for N concentration and δ ^15^N isotopic concentration (Isotope Tracer Technologies Inc., Ontario, Canada). Grain yield measurements served as a basis for assessing the impact of BNF on the crop's response to synthetic N fertilizer. End-of-season grain yields (adjusted to 15.5% moisture content) were obtained by harvesting the central two rows of each plot using a small combine. We employed a quadratic-plateau nonlinear regression approach to derive three critical parameters of the N response curve: the yield without N fertilizer (*a*), the initial slope of the yield response (*b*), and the maximum attainable yield (*Y*_*max*_). We then tested whether the inoculation treatment (NTC vs. PROVEN 40), the trial year (2021 and 2022), or their interaction influenced these curve parameters. The computed model parameters were used to determine the agronomic optimum N fertilizer rate (AONR) for each combination of year and treatment with bootstrapped 95% confidence intervals for the response curves and AONR^54^. More details are shown in the supplemental information (supplemental text S2).

#### Inoculation rate trials

Twenty-nine small plot trials were conducted in 2022 by Pivot Bio, Inc. through contract research organizations. Experiments were set up as strip trials following a RCB design with 6 replicates. Treatments included: 1) uninoculated control with the local business-as-usual (BAU) N fertilizer rate; 2) uninoculated control where N fertilizer rate was reduced by 44.8 kg N ha^-1^; 3) and three inoculation rate treatments (Low, Medium and High) with reduced N fertilizer rates. Further details on the management at each trial are shown in the supplemental Table S7.

In the inoculation rate treatments, maize seeds were inoculated prior to planting with commercial-grade inoculant with Kv137-2253 and Ks6-5687 strains (PROVEN 40) by soaking seeds in the resuspended liquid formulation containing both live strains at various concentrations. Inoculation rates of the combined strains were roughly 10^4^, 10^5^ and 10^6^ CFU per seed. We expected cell viability to decline by about a factor of 10 by planting time (up to 45 days post inoculation). We assessed inoculum cell viability at planting of a subset of trials and determined it adequately reflected the desired inoculation rate ladder (supplemental Fig. S2).

##### Plant sampling

Plot plant population was assessed at around the V2 stage by counting emerged plants in a section of the center 2 rows within a standard length (typically ~ 5 m). At around the V4-V5 growth stages, root ball samples were collected from two non-adjacent maize plants by uprooting using a shovel to retrieve the root to a depth of 30–45 cm. Excess soil was removed by shaking gently, and aerial tissues were removed by cutting the stalk just above the crown. To minimize cross contamination, gloves and shovels were cleaned or changed between plots. Unwashed root samples were shipped to the lab in cold (4 ⁰C) for colonization analysis. At the VT-R1 growth stage, representative plants were collected from each plot by cutting at the soil level, chopped to 10 cm sections and sent to laboratory to determine dry mass and N concentration (Waypoint Analytica LLC, Memphis, TN, U.S.). Aboveground biomass N at VT-R1 was estimated as the product of plant dry mass and N concentration, scaled by the plot plant population (in kg N ha^-1^). A subsample of the ground (< 1 mm), homogenized plant biomass tissue was sent aside for ^15^N isotope concentration analysis by a third-party laboratory (Isotope Tracer Technologies Inc., Ontario, Canada). Note that V4-V5 root samples and VT-R1 aboveground biomass samples were collected only in a subset of sites (supplemental Table S7). Grain yield was determined after grain moisture had dropped below 20% by harvesting the center four rows of the plot using a mechanical combine harvester. Yields are reported adjusted to 15.5% moisture. Aboveground biomass N, grain yields and δ^15^N concentration metrics were tested for the null hypothesis of no treatment differences using a linear mixed-effect model with treatment as the fixed effect and replicate within site as the random effect. If appropriate, measurements were log-transformed to correct for heteroscedasticity and back-transformed to report results.

##### Field colonization

To quantify the abundance of the target microbial strains in the V4-V5 root samples, we employed a quantitative PCR (qPCR) approach. Genomic DNA (gDNA) was extracted using the following methods. Roots were lightly rinsed to remove bulk soil. Then tissues from a "standard sample" zone (seminal to node 3, ~ 2.5 cm below the seed) were cut into 1 cm pieces and weighed directly into a 24-well plate containing 12.7 mm steel homogenization beads. Root samples (0.5–0.6 g) were then frozen at -80 ⁰C, homogenized using a Spex genogrinder (Cole-Parmer, Metuchen, NJ, U.S.). gDNA was extracted using a custom mix of AT/ATL lysis buffers (QIAGEN GmbH, Hilden, Germany; PN: 19,075, 939,016) and elution buffers (Zymo Research Corp., Irvine, CA, U.S.; PN: D3004-2–400, D3004-4–50). The qPCR reaction was performed using the MDX020 Probe Low Rox kit (Meridian Biosciences Inc., Cincinnati, OH, U.S.) in an Applied Biosystems Quant 7 Thermocycler (Thermo Fisher Scientific Inc., Waltham, MA, U.S.). Amplification primers were designed in-house to target the edits or gene regions that we found to be reasonably unique to Ks6-5687 and Kv137-2253. The amplified products were quantified via dye fluorescence, which then was used to estimate abundance of the target sequences. qPCR readings were expressed as copies per g of root fresh tissue. Following *ref.*^55^ we analyzed these data using a zero-inflated gaussian mixed-effect model approach where the dependent variable is transformed with *log(y* + *1)* to control for heteroskedasticity while retaining zero-inflation. Inoculation rate and target strain were set as the fixed effects, while replicate within trial as random intercepts.

#### Statistical analysis of field ^15^N measurements

Plant samples from the ^15^N enrichment-dilution trials and ^15^N natural abundance trials were used to compare the ^15^N abundance in the biomass of plants inoculated with PROVEN 40 to the uninoculated control (NTC) and estimate the percent of aboveground biomass N derived from the atmosphere (Ndfa). When depending on natural ^15^N abundance across many sites, sites were weighed according to the mean δ^15^N of the reference plots as we expect low levels of background δ^15^N to limit detection of δ^15^N dilution. All field ^15^N isotope experiments were analyzed by fitting linear mixed-effects models with fixed and random effects per the experimental design. If models failed assumptions of normality or homoscedasticity according to the Shapiro–Wilk or Levene tests, respectively, or by examination of residual-by-predicted scatter plots, models were refit with log-transformed data and reevaluated for improved fit. When higher-order interactions were not significant, they were removed, models were re-fit, and the improved fit was evaluated using the Akaike information criterion (AIC). Additional details of linear mixed-effects model fitting are provided in supplemental Table S9. All tests of hypotheses of δ^15^N data were conducted using α = 0.1.

Percent Ndfa was calculated as the change from control δ^15^N abundance, divided by the marginal mean δ^15^N abundance of the control, assuming the $\updelta {\text{N}}_{air}$ is zero using the following equation:

1 $$\%Ndfa=100* \left(\frac{\delta^{15}N_{NTC}-\delta^{15}N_{PROVEN40}}{\delta^{15}N_{NTC}-\delta^{15}N_{air}}\right)$$

### Commercial-scale trials

Large-scale, nonreplicated, side-by-side demonstration trials were established in commercial maize fields throughout the United States during 2021, 2022, and 2023. Cooperator growers were voluntarily enrolled through a commercial incentive program. Each field consisted of two treatments: i) business-as-usual (BAU) treatment that followed each grower’s standard practices, and ii) a modified practice where growers reduced synthetic N fertilizer inputs (by 39 to 45 kg N ha^-1^) and applied PROVEN 40. The growers determined the trial layouts. Yield maps were collected in 2021, and early-season plant traits were measured in 2022 and 2023 from a subset of trials.

#### On-farm plant response

Maize plants were sampled during the V6 to VT growth stages of fields in 2022 and 2023 from a total of 135 locations. Six plants were randomly selected and removed from each treatment zone at each site by cutting at the soil surface. Plant aboveground fresh weight (FW) was measured immediately using a hanging scale (AWS-SR-5, American Weigh Scales, Atlanta, Georgia, U.S.) and a bucket. The chlorophyll content index (CCI) of the uppermost collared leaf was measured using an MC-100 Chlorophyll Concentration Meter (Apogee Instruments, Logan, Utah, U.S.), with four measurement points along the middle of each leaf, equidistant between the leaf edge and midrib, which were then averaged. Prior to statistical analysis, CCI was converted to chlorophyll concentration (CC; µmol m^-2^), using the following equation^56^:

2 $$CC=-121+129*(CCI)^{0.42}$$

The null hypothesis of no treatment differences in aboveground fresh weight and leaf chlorophyll concentration was evaluated using a nonparametric two-sided paired t-test or a Wilcoxon signed-rank test with continuity correction if the distribution of the differences failed a Shapiro–Wilk normality test with a *p* < 0.05.

To validate the relationship between total aboveground N (kg ha^-1^) and measurements of aboveground fresh weight and chlorophyll concentration, we conducted additional plant sampling in a subset of these fields (n = 9) at stages ranging from V7 to VT. Individual plants (n = 18 plants per treatment) were collected, fresh weight and leaf chlorophyll concentration were measured as described above, and subsamples of two immediately adjacent plants were sent to the laboratory for drying, weighing, and tissue N concentration analysis (Midwest Labs, Atlantic, Iowa, U.S.). A predictive model was developed by regressing these field measurements against the log-transformed aboveground biomass N. The model was then cross-validated by iteratively leaving one site out each time and refitting the model, and the full model was used to predict percent changes in aboveground biomass N across all sites. More details about model development and testing are available in the supplemental information (supplemental Fig. S4).

#### On-farm yield response

In 2021, yield monitor datasets were acquired from 58 grower fields through a third-party farm data software manager (INTENT, https://intent.ag/). Growers submitted yield and ancillary datasets through INTENT’s data ingestion portal, which subsequently were cleaned and moisture-corrected (15.5% moisture) by the software and spatially joined with plot-treatment information. Growers applied the treatment (39–45 kg ha^-1^ less than BAU with PROVEN 40) across one or more strips in their fields. The size and number the strips varied among locations, with an average area of 11.3 ha of treated area by location. For the BAU treatment area, we selected an equally sized area adjacent to each strip. This aimed to represent a side-by-side comparison at each location. (An example of the setup is shown in the supplemental Fig. S5). Yield was summarized at the site level using the mean and coefficient of variation. Both site-level metrics were tested for the null hypothesis of no treatment differences using a linear mixed-effect model with treatment as the fixed effect and site as the random effect. The coefficient of variation was log-transformed to correct for heteroscedasticity and back-transformed to report results. Minimum detectable effects were calculated using the standard error of the estimated treatment contrast and the expected t-ratio (two-tailed with α = 0.05).

### Statistical software

All statistical analyses were performed and documented with Jupyter Notebooks^57^using the R statistical software (version 4.1.3)^58^ kernel augmented with the following packages and their dependencies:

- Data munging and visualization: *tidyverse, readxl, broom, foreach*, *ggpubr, ggthemes, scales, png*
- Model fitting and evaluation: *lme4, lmerTest, nlme, glmmtmb, car, DHARMa, caret*
- Post-hoc mean comparisons: *emmeans, multcomp*

A complete list of all attached packages and their citations is provided in the supplemental information.

## Supplementary Information

Supplementary Information.

## Data Availability

The datasets and computer analysis code used to support the findings of this study are available in the following repository: https://doi.org/10.5281/zenodo.10658648.
