# Supplementary material for: Genetic remodeling of soil diazotrophs enables partial replacement of synthetic nitrogen fertilizer with biological nitrogen fixation in maize
Source: Sci Rep. 2024 Nov 12;14:27754. doi: 10.1038/s41598-024-78243-3 (PMC11557888; doi:10.1038/s41598-024-78243-3)
Supplement: Supplementary file 1 — Supplementary Information. [file 41598_2024_78243_MOESM1_ESM.docx]

**Supplemental information to:**

Genetic remodeling of soil diazotrophs enables partial replacement of synthetic nitrogen fertilizer with biological nitrogen fixation in maize

*Rafael Martinez-Feria ^1,5^*, Maegen B. Simmonds ^1,2,5^*, *Bilge Ozaydin ^1^, Stacey Lewis ^1^, Allison^1^ Schwartz, Alex Pluchino ^1^, Megan McKellar ^1^, Shayin S. Gottlieb ^1^, Tasha Kayatsky ^1^, Richelle Vital ^1^, Sharon E. Mehlman ^1^, Zoe Caron ^1^, Nicholas R. Colaianni ^1^, Jean-Michel Ané ^3^, Junko Maeda ^3^, Valentina Infante ^3^, Bjorn H. Karlsson ^3^, Caitlin McLimans ^3^, Tony Vyn ^4^, Brendan Hanson ^4^, Garrett Verhagen ^1,4^, Clayton Nevins¹, Lori Reese¹, Paul Otyama¹, Alice Robinson¹, Timothy Learmonth¹, Christine M. F. Miller¹, Keira Havens ¹, Alvin Tamsir ¹, Karsten Temme¹*

*^1^ Pivot Bio, Inc. 2910 Seventh St, Berkeley, CA 94710 2*

*^2^ Now at: Regrow Agriculture, Inc., Durham, NH 03824*

*^3^ Department of Plant and Agroecosystem Sciences, University of Wisconsin-Madison, 1575 Linden Drive, Madison, WI 53706*

*^4^ Department of Agronomy, Purdue University, 915 Mitch Daniels Blvd, West Lafayette, IN 479074*

*^5^Joint lead authorship*

**Corresponding author (*[*rmartinez-feria@pivotbio.com*](mailto:rmartinez-feria@pivotbio.com)*)*

## Supplemental Text S1: Details on genetic modifications and strain remodeling

In previous studies (Wen *et al* 2021, Bloch *et al* 2020), *Klebsiella variicola* (*Kv*137) and *Kosakonia sacchari* (*Ks*6), both gamma-proteobacteria, were isolated and characterized for their diazotrophic properties. These strains are known for their robust colonization of the maize rhizosphere and well-characterized BNF-related genes *(nif* regulon). To enhance their BNF activity under N-rich conditions and promote ammonium release into the extracellular environment, we rationally engineered these strains through non-transgenic methods, resulting in modified strains referred to as Kv137-2253 and Ks6-5687.

To boost nitrogenase protein activity in both strains, we first edited the *nifLA* operon by deleting the *nifL* inhibitor and inserting a strong, native promoter upstream of the *nifA* gene, using the suicide plasmid mutagenesis method (Selvaraj and Iyer 1983). We copied a sequence containing the promoter for the *ompX* gene and the promoter for the *infC* gene into *Ks*6-5687 and *Kv*137-2253, respectively. This edit allowed for the constitutive expression of *nifA* under N-rich conditions (Fig 1b in main text). Then we pursued multiple approaches to facilitate the release of fixed N for plant benefit (Fig 1c in main text). One approach involved editing the GlnD protein, which plays a role in sensing cellular N levels through glutamine. Using suicide plasmid mutagenesis, we deleted the entire *glnD* gene (*Ks*6-5687, *ΔglnD*) or eliminated its uridylyl transferase domain (*Kv*137-2253, *glnD-UTase*) to decrease ammonium assimilation through GlnD regulation. Additionally, in Ks6-5687, we edited the *glnE* gene, which regulates glutamine synthetase (GlnA) activity. By removing the adenylyl-removal domain of *glnE*, we kept GlnA in an adenylated inactive state, leading to increased ammonium excretion in *Ks*6-5687 to a similar level to that of *Kv*137-2253. These modifications aimed to enhance BNF activity and ammonium release in these strains for improved plant symbiosis. We refer the reader to *refs.* (Wen *et al* 2021, Bloch *et al* 2020) for more details on the genetic edits.

**Supplemental Table S1a. Description of strains and genetic remodeling.**

| Species | Strain | Genotype | Phenotype |
| --- | --- | --- | --- |
|  | | | |
| *Kosakonia sacchari* | *Ks*6-5687 | *ΔnifL::PompX-nifA, ΔglnD, glnE-ΔAR_rebuild* | Derepressed, Enhanced Excreting |
|  | *Ks*6-7023 | *ΔnifL::PompX-nifA, ΔglnD, glnE-ΔAR_rebuild, ΔnifH* | *nif*KO |
|  | *Ks*6 | Wildtype | Wildtype |
|  | | | |
| *Klebsiella variicola* | *Kv*137-2253 | *ΔnifL::PinfC-nifA, glnD-ΔUTas*e | Derepressed, Enhanced Excreting |
|  | *Kv*137-1036 | *ΔnifL::PinfC-nifA* | Derepressed, Excreting |
|  | *Kv*137-7036 | *ΔnifL::PinfC-nifA, glnD-ΔUTase, ΔnifH* | *nif*KO |
|  | *Kv*137 | Wildtype | Wildtype |

**Supplemental Table S2. Detailed summary of the experimental setup for the trials conducted** **during the 2021 and 2022 growing seasons at the Hancock and Arlington Agricultural Research Stations of the University of Wisconsin-Madison to evaluate and quantify the delivery of fixed N from the remodeled bacterial strains to maize plants using the ^15^N enrichment-dilution method.**

| **Location** | **Hancock, WI - 2021** | **Hancock, WI - 2022** | **Arlington, WI - 2022** |
| --- | --- | --- | --- |
| **Latitude, Longitude** | 44°7, -89°32’ | 44°7, -89°32’ | 43°18’, -89°21’ |
| **Soil Type** | Plainfield sand | Plainfield sand | Plano silt loam |
| **Soil pH** | 5.9 | 6.4 | 6.7 |
| **Soil Organic Matter** | 0.6% | 0.6% | 2.8% |
| **Soil Water-Holding Capacity (top 1 m)** | 35 mm | 35 mm | 184 mm |
| **Previous Crop** | Maize | Maize | Maize |
| **Experimental Design** | RCBD with 12 replications | RCBD with 30 replications | RCBD with 30 replications |
| **Experimental Unit** | Three rows x 3 m, each with 17 plants | Three rows x 3 m, each with 17 plants | Three rows x 3 m, each with 17 plants |
| **Planting Date** | 12-May-21 | 26-May-22 | 31-May-22 |
| **Maize Hybrid** | PHJ89 x PH207 | PHJ89 x PH207 | PHJ89 x PH207 |
| **Bacterial Inoculation (Seeds)** | At planting | At planting | At planting |
| **Bacterial Inoculation (Roots)** | V2 | V2 | V2 |
| **Root Colonization Sampling** | V5 | V5 | V5 |
| **Control Treatment** | PBS | PBS | PBS |
| **Enriched Fertilizer Schedule** |  |  |  |
| **Pre-plant** | 11-May, 22 kg N/ha ATS with 1% 15N ^1^ | 5-May, 22 kg N/ha ATS with 1% 15N | none |
| **First Side Dress** | 4-Jun, 35.3 kg N/ha urea with 1% 15N | 10-Jun, 35.3 kg N/ha urea with 1% 15N | 2-Jun, 112 kg N/ha AMS with 1% 15N |
| **Second Side Dress** | 21-Jun, 64.4 kg N/ha UAN with 1% 15N | 24-Jun, 64.4 kg N/ha UAN with 1% 15N | 28-Jun, 34.7 kg N/ha AMS with 1% 15N |
| **Third Side Dress** | 29-Jun, 59.5 kg N/ha UAN with 1% 15N | 8-Jun, 59.5 kg N/ha UAN with 1% 15N | 21-Jul, 34.7 kg N/ha AMS with 1% 15N |
| **Fourth Side Dress** | 17-Jul, 59.5 kg N/ha UAN with 1% 15N | 20-Jul, 59.5 kg N/ha UAN with 1% 15N | none |

**Footnotes:**

^1^ The fertilizer was enriched to 1.0 atom % ^15^N using ammonium sulfate (98 atom % ^15^N, Millipore Sigma, item# 299286) applied as a powder or liquid solution and incorporated uniformly into the soil.

**Supplemental Table S3. Detailed summary of the experimental setup for the trials conducted at the Purdue University Agronomy Center for Research and Education (ACRE) farm.**

| **Year** | **2021** | **2022** |
| --- | --- | --- |
| **Location** | West Lafayette, IN | West Lafayette, IN |
| **Latitude, Longitude** | 40.477413°, -87.004443° | 40.4700291°, -86.9917576° |
| **Soil Type** | Chalmers silty clay loam | Raub-Brenton complex, 0 to 1 percent slopes |
| **Soil pH** | 6.9 | 6.2 |
| **Soil Organic Matter** | 4.1% | 3.7% |
| **Soil Water-Holding Capacity (top 1 m)** | 172 mm | 157 mm |
| **Previous Crop** | Soybean | Soybean |
| **Experimental Design** | Split-Plot 8 reps per treatment, with N rate as main plot, and inoculation as subplot | Split-Plot 8 reps per treatment, with N rate as main plot, and inoculation as subplot |
| **Plot Size (Experimental Unit)** | 4.6 (6-row) x 22.9 m | 4.6 (6-row) x 27.4 m |
| **Inoculation Treatment** | Commercial grade inoculant of Ks6-5687 + Ks137-2253 (PROVEN 40) at 0.9 L/ha applied at planting | Commercial grade inoculant of Ks6-5687 + Ks137-2253 (PROVEN 40) at 0.9 L/ha applied at planting |
| **Control Treatment** | None | None |
| **Pre-plant Fertility** | Spring ATS (66 L/ha, 10 kg N/ha) | Fall Potash (186 kg K/ha), Spring ATS (66 L/ha, 10 kg N/ha) |
| **Spring Tillage Post Pre-plant Fertility** | Cultivator, 20.3 cm depth | Cultivator, 20.3 cm depth |
| **Planting Date** | 4/28/2021 | 5/2/2022 |
| **Hybrid** | P1359AM | P1359AM |
| **Target Population** | 7.7 plant m^-2^ | 8.4 plant m^-2^ |
| **Emergence Date** | 5/12/2021 | 5/13/2022 |
| **N Side Dress Date** | 6/2/2021 | 5/31/2022 |
| **N Side Dress Rates** | 0, 106, 151, 186, 241, 286 kg N/ha as 28% UAN coulter injected ~15 cm | 0, 106, 151, 186, 241, 286 N/ha as 28% UAN coulter injected ~15 cm |
| **V8 Sampling Date** | 6/14/2021 | 6/14/2022 |
| **V8 Partitions** | Whole Plant, Leaf Tip | Whole Plant, Leaf Tip |
| **R1 Sampling Date** | 7/19/2021 | 7/18/2022 |
| **R1 Partitions** | Leaf, Stem, Ear | Leaf, Stem, Ear |
| **R6 Sampling Date** | 9/21/2021 | 9/23/2022 |
| **R6 Partitions** | Leaf, Stem, Cob, Grain | Leaf, Stem, Cob, Grain |
| **Harvest Date** | 9/29/2021 | 10/6/2022 |
| **Harvest Area** | 34.8 m^2^ (Kincaid 2-row Combine) | 41.8 m^2^ (Kincaid 2-row Combine) |

| 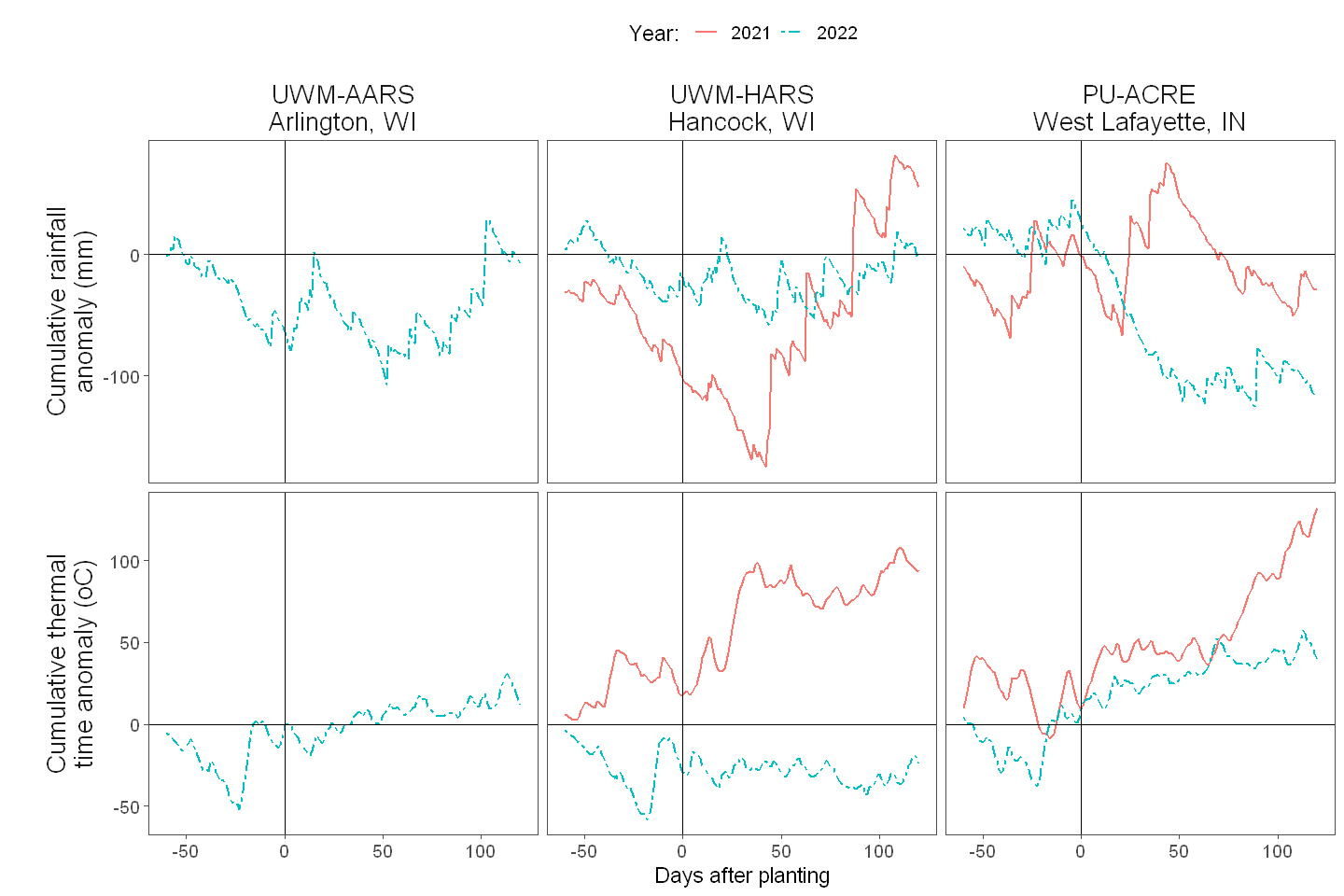 |
| --- |
| **Supplemental Figure S1. Weather conditions at the ^15^N enrichment-dilution (Arlington, Hancock) and ^15^N natural abundance (ACRE) field trials.** Weather source: DAYMET (Thornton *et al* 2022). |

**Supplemental Table S4. Estimated aboveground biomass N uptake and total shoot N derived from the atmosphere (Ndfa) at flowering (VT) in plots inoculated with PROVEN 40 at the Purdue University Agronomy Center for Research and Education (ACRE) farm experiments.**

| Year | 2021 | 2022 |
| --- | --- | --- |
| Aboveground biomass N  (kg N ha^-1^) | 189.7 (±4.785)^1^ | 102.6 (±2.646) |
| Ndfa  (%) | 10.2 (±4.32) | 13.6 (±7.36) |
| Ndfa  (kg N ha^-1^) | 19.42 (±8.21)^2^ | 13.95 (±7.557) |

**Footnotes:**

^1^ Values within parentheses correspond to the standard error of the estimates.

^2^ Error for total Ndfa in kg N ha^-1^ was propagated using $y=(\hat{a}*\hat{b} )\sqrt[2]{\left( \frac{a_{SE}}{\hat{a}} \right)^{2}+\left( \frac{b_{SE}}{\hat{b}} \right)^{2}}$, where $\hat{a}$ and $\hat{b}$ are the estimates for Aboveground biomass N and Nfda (%), and $a_{SE}$ and $b_{SE}$ are their respective standard errors.

## Supplemental text S2: Details on yield response to N fertilizer and AONR estimation at Purdue University ACRE farm trials

The yield measurements collected from the N rate trials at Purdue University in 2021 and 2022 were analyzed to evaluate the effect of microbially fixed N from PROVEN 40 inoculations on the crop’s response to synthetic N fertilizer. For this, we employed a nonlinear regression modeling approach in which the yield response to the N fertilizer rate was hypothesized to follow a quadratic-plateau curve in the form:

$$Y\left( N_{f} \right)=\left( a+bN_{f}+cN_{f}^{2} \right)*\left( N_{f}\leq0.5\frac{b}{c} \right)+\left( a+\frac{-b^{2}}{4c} \right)*\left( N_{f}>0.5\frac{b}{c} \right)$$

(Eq. S1)

where $N_{f}$is the N fertilizer rate, $Y\left( N_{f} \right)$ is the yield as function Nf, $a$ is the yield with zero N fertilizer, $b$ is the slope of the response at $N_{f}$= 0, and $c$ is a shape parameter. Inspection of this expression makes evident that the plateau is reached when $N_{f}=0.5\frac{b}{c}$ . This critical point, commonly referred to as the Agronomic Optimum N Rate (AONR), represents the N fertilizer level beyond which yield no longer responds to fertilizer N additions. This maximum yield level ($Y_{max}$) is calculated as:

$$Y_{max}= a+\frac{-b^{2}}{4c}$$

(Eq. S2)

For ease of interpretability, we can solve for *c* in Eq. S2 and substitute in Eq. 1 to expose $Y_{max}$as one of the parameters in the model:

$$Y\left( N_{f} \right)= \left( a+bN_{f}+ \frac{-b^{2}}{4 \left( Y_{max}-a \right)}N_{f}^{2} \right)*\left( N_{f}\leq\frac{-2\left( Y_{max}-a \right)}{b} \right)+ Y_{max}*\left( N_{f}>\frac{-2\left( Y_{max}-a \right)}{b} \right)$$

(Eq. S3)

We fitted a nonlinear mixed-effect model using Eq. S3 as the null model with $N_{f}$ as the independent variable, $Y\left( N_{f} \right)$ as the dependent variable, $a,b$ and $Y_{max}$ as curve parameters (fixed effects), and replicated within trial year as the random effect. We additionally tested whether the inoculation treatment (*trt*; NTC vs PROVEN 40) and trial year (2021 and 2022), and their interaction affected any of the curve parameters by including them as fixed effects (full model).

To protect against overparameterization, we also tested progressively more parsimonious configurations of fixed effects as shown in supplemental Table S7. We then selected the model configuration that best fit the data, as characterized by the model with the smallest AIC value. All model fits were performed using the *nlme* library in R (version 4.1.2).

After selecting the model configuration with the lowest AIC value (Model 3), we proceeded to check for signs of model assumption violations using graphical diagnostic methods (residual plots, quantile-quantile plots), and then conducted tests of hypothesis for all fixed parameters, using an alpha of 0.05. Finally, following the methodology of Francis et al., (2021), we computed 95% confidence intervals for the response curves and AONR values using residual-resampling bootstrapping, where the model residuals were resampled with replacement 300 times to generate a population of parameters for computing distributions of AONR and responses.

**Supplemental Table S5.** **Configurations of fixed effects of competing non-linear quadratic plateau models in response to grain yield (Mg ha^-1^) and their interpretations.**

| Model | Fixed Effects | Interpretation | AIC |
| --- | --- | --- | --- |
| Full | a + b + Y_max_ ~ trt*year | Interactive effect of *trt* or *year* on all curve parameters | 567.625 |
| Model 1 | a ~ trt*year  b + Y_max_ ~ trt + year | Interactive effects on *a*, and both main effects on *b* and *Y_max_* | 563.906 |
| Model 2 | a + b + Y_max_ ~ trt + year | Main effects of *trt* or *year* on all curve parameters | 565.576 |
| Model 3 | a ~ trt*Year  b + Y_max_ ~ year | Interactive effects on *a*, and only year effects on *b* and *Y_max_* | 560.64 |
| Model 4 | a ~ trt + Year  b + Y_max_ ~ year | Main effects on *a*, and main effect of year on *b* and *Y_max_* | 562.424 |
| Model 5 | a ~ trt  b + Y_max_ ~ year | Main effects on a, and main effect of year on *b* and *Y_max_* | 561.301 |
| Model 6 | a + b + Y_max_ ~ year | Only year main effects | 571.685 |
| Null | a + b + Y_max_ ~ 1 | No effect of *trt* or *year* on curve parameters | 576.485 |

**Supplemental Table S6.**  Parameter estimates for nonlinear quadratic plateau mixed-effects models of the grain yield response to N fertilizer for inoculation treatments (Trt) at two contrasting site years (Yr).

(a) Null Model

|  |  | **Fixed effects** | | | | **Random effects** | | **Residual** |
| --- | --- | --- | --- | --- | --- | --- | --- | --- |
| **Parameter** | **Statistic** | **Intercept** | **Yr** | **Trt** | **Yr * Trt** | **Yr** | **Rep within Yr** |  |
| **a** | Estimate | 8.00 | - | - | - | - | - | 0.8437 |
|  | SE | 0.232 | - | - | - | - | - |  |
|  | p-value | < .001 | - | - | - | - | - |  |
|  | Std. deviation | - | - | - | - | 1.3x10^-4^ | 0.71 |  |
| **b** | Estimate | 0.064 | - | - | - | - | - |  |
|  | SE | 0.007 | - | - | - | - | - |  |
|  | p-value | <.001 | - | - | - | - | - |  |
|  | Std. deviation | - | - | - | - | 0.00856 | 0.0114 |  |
| **Y_max_** | Estimate | 16.5 | - | - | - | - | - |  |
|  | SE | 0.689 | - | - | - | - | - |  |
|  | p-value | < .001 | - | - | - | - | - |  |
|  | Std. deviation | - | - | - | - | 0.864 | 1.065 |  |

(b) Full Model

|  |  | **Fixed effects** | | | | **Random effects** | | **Residual** |
| --- | --- | --- | --- | --- | --- | --- | --- | --- |
| **Parameter** | **Statistic** | **Intercept** | **Yr** | **Trt** | **Yr * Trt** | **Yr** | **Rep within Yr** |  |
| **a** | Estimate | 7.72 | -0.19 | 0.96 | -0.43 | - | - | 0.799 |
|  | SE | 0.38 | 0.54 | 0.40 | 0.57 | - | - |  |
|  | p-value | < .001 | 0.72 | 0.02 | 0.45 | - | - |  |
|  | Std. deviation |  |  |  | - | 3.38x10^-5^ | 0.695 |  |
| **b** | Estimate | 0.07 | -0.01 | -1.7 x10^-4^ | 0.00 | - | - |  |
|  | SE | 0.01 | 0.01 | 0.01 | 0.01 | - | - |  |
|  | p-value | < .001 | 0.07 | 0.98 | 0.56 | - | - |  |
|  | Std. deviation |  |  |  | - | 7.72x10^-7^ | 0.0107 |  |
| **Y_max_** | Estimate | 17.37 | -1.91 | 0.13 | 0.02 | - | - |  |
|  | SE | 0.44 | 0.63 | 0.31 | 0.48 | - | - |  |
|  | p-value | < .001 | < .001 | 0.68 | 0.97 | - | - |  |
|  | Std. deviation | - | - | - | - | 6.28x10^-5^ | 0.985 |  |

(c) Reduced model (Model 3)

|  |  | **Fixed effects** | | | | **Random effects** | | **Residual** |
| --- | --- | --- | --- | --- | --- | --- | --- | --- |
| **Parameter** | **Statistic** | **Intercept** | **Yr** | **Trt** | **Yr*Yrt** | **Yr** | **Rep within Yr** |  |
| **a** | Estimate | 7.710 | -0.059 | 0.990 | -0.700 | - | - | 0.802 |
|  | SE | 0.349 | 0.491 | 0.267 | 0.369 | - | - |  |
|  | p-value | 0.000 | 0.904 | 0.000 | 0.059 | - | - |  |
|  | Std. deviation | - | - | - | - | 3.43x10^-5^ | 0.69 |  |
| **b** | Estimate | 0.072 | -0.017 | - | - | - | - |  |
|  | SE | 0.005 | 0.007 | - | - | - | - |  |
|  | p-value | 0.000 | 0.017 | - | - | - | - |  |
|  | Std. deviation | - | - | - | - | 5.79x10^-7^ | 0.011 |  |
| **Y_max_** | Estimate | 17.448 | -1.900 | - | - | - | - |  |
|  | SE | 0.403 | 0.574 | - | - | - | - |  |
|  | p-value | 0.000 | 0.001 | - | - | - | - |  |
|  | Std. deviation | - | - | - | - | 5.34x10^-5^ | 0.981 |  |

**Supplemental Table S7. Details on site characteristics for the inoculation rate trials.**

| **Trial** | **Measurements^1^** | | | | **Trial Description** | | | | | | | | | | | | | | |
| --- | --- | --- | --- | --- | --- | --- | --- | --- | --- | --- | --- | --- | --- | --- | --- | --- | --- | --- | --- |
|  | **QPCR**  **@ V4-V5** | **δ^15^N @ VT-R1** | **Biomass @ VT-R1** | **Yield @ harvest** | **Lat. (deg)** | **Long. (deg)** | **Irrigated** | **OM^2^ (%)** | **Clay^2^ (%)** | **Sand^2^ (%)** | **Planting date (M/D/Y)** | **Mean temp.^3^ (◦C)** | **Rain^3^ (mm)** | **Previous crop** | **Maize hybrid** | **Relative maturity (days)** | **Plant density (p/m^2^)** | **N fert. rates ^4^**  **(kg N/ha)** | **N fert. Management ^5^** |
| CO01451 |  |  | X | X | 40.5 | -105 | Yes | 1.7 | 26 | 55 | 5/17/2022 | 21.3 | 133 | Maize | N/A | 100 | 8.38 | 230, 185 | Preplant UAN + postplant top-dressed urea (45 kg N/ha, only in BAU) |
| IA01451 |  |  | X | X | 42.1 | -93.5 | No | 3.9 | 26 | 51 | 5/22/2022 | 20.8 | 630 | Maize | DKC61-41RIB | 111 | 8.42 | 185, 140 | At-plant UAN |
| IA02451 |  | X | X | X | 41.2 | -94.9 | No | 3.55 | 30 | 37 | 5/12/2022 | 21 | 406 | Soybean | DKC61-41RIB | 111 | 8.43 | 213, 168 | Fall AA + At-plant UAN (45 kg N/ha, only in BAU) |
| IA03451 | X |  | X | X | 43.2 | -95.6 | No | 5.2 | 34.5 | 27.5 | 4/27/2022 | 19 | 380 | Soybean | DKC52-18RIB | 102 | 8.4 | 218, 173 | Fall DAP (62 kg N/ha) + postplant UAN |
| IA04451 | X | X | X | X | 42.1 | -93.6 | No | 3.1 | 25 | 49 | 5/19/2022 | 21.2 | 572 | Soybean | N/A | 110 | 8.1 | 196, 151 | At-plant UAN |
| IL01451 | X |  | X | X | 41.5 | -89.6 | No | 4.9 | 21 | 31 | 5/11/2022 | 20.3 | 531 | Soybean | DKC52-18RIB | 102 | 8.1 | 196, 151 | At-plant UAN |
| IL03451 | X | X | X | X | 40.9 | -89.5 | Yes | 2.25 | 20 | 54 | 5/23/2022 | 22.6 | 416 | Soybean | DKC52-18RIB | 102 | 8.13 | 212, 167 | Preplant MAP (13 kg N/ha) + at-plant UAN + sidedress UAN (45 kg N/ha, only in BAU) |
| IN01451 | X |  | X | X | 40.2 | -86.6 | No | 3.4 | 32 | 40.5 | 5/30/2022 | 21.1 | 426 | Soybean | P1082AM | 110 | 8.63 | 224, 179 | At-plant UAN |
| IN02451 | X |  | X | X | 40.1 | -85.9 | No | N/A | N/A | N/A | 5/17/2022 | 21.6 | 465 | Soybean | P1082AM | 110 | 7.64 | 190, 145 | At-plant urea |
| KY01451 |  |  |  | X | 38.4 | -94.7 | No | 4.1 | 33 | 22 | 6/14/2022 | 25 | 397 | Soybean | P1082AM | 110 | 8.05 | 224, 179 | Spring AA |
| MI01451 | X | X | X | X | 37.5 | -87.3 | No | N/A | N/A | N/A | 5/30/2022 | 24.8 | 550 | Soybean | DKC61-41RIB | 111 | 7.74 | 168, 112 | Spring urea |
| MN01451 |  | X | X | X | 42.4 | -84.8 | No | 1.65 | 19 | 59 | 5/20/2022 | 19.7 | 447 | Soybean | DKC52-18RIB | 102 | 6.7 | 168, 123 | Postplant UAN |
| MN02451 |  | X | X | X | 46.8 | -96.6 | No | 4.6 | 32 | 32 | 5/24/2022 | 19.9 | 337 | Soybean | DKC31-10RIB | 81 | 7.81 | 190, 140 | At-plant urea, MAP |
| MN03451 |  |  | X | X | 45.3 | -95.3 | No | 5.25 | 26 | 35 | 6/2/2022 | 18.5 | 354 | Maize | P0075AM | 100 | 8.42 | 170, 125 | Preplant urea, AMS, MAP + at-plant urea (45 kg N/ha, only in BAU) |
| MO01451 |  | X | X | X | 39.6 | -95 | No | 2.4 | 22 | 45 | 5/10/2022 | 22.2 | 498 | Soybean | DKC61-41RIB | 111 | 8.13 | 196, 151 | Spring AA + at-plant UAN (45 kg N/ha, only in BAU) |
| MO02451 |  |  | X | X | 36.7 | -90.2 | Yes | 2.45 | 21 | 25 | 5/12/2022 | 24.9 | 405 | Maize | DKC61-41RIB | 111 | 8.73 | 220, 175 | At-plant MAP + postplant top-dressed urea (45 kg N/ha, only in BAU) |
| MS01451 | X | X | X | X | 33.3 | -90.9 | Yes | N/A | N/A | N/A | 4/27/2022 | 25.5 | 443 | Soybean | DKC61-41RIB | 111 | 8.02 | 196, 151 | At-plant UAN |
| NC01451 |  | X |  |  | 35.7 | -76.7 | No | N/A | N/A | N/A | 4/15/2022 | 24 | 639 | Maize | DKC52-18RIB | 102 | 6.9 | N/A | N/A |
| ND01451 |  | X | X | X | 48.2 | -101 | No | 4.4 | 26 | 41 | 5/24/2022 | 18.9 | 236 | Soybean | DKC31-10RIB | 81 | 9.41 | 135, 90 | N/A |
| ND02451 |  | X | X | X | 47.7 | -97.6 | No | 3.35 | 18 | 62 | 5/26/2022 | 19 | 300 | Soybean | DKC31-10RIB | 81 | 8.25 | 146, 101 | At-plant urea, MAP |
| NE01451 |  |  | X | X | 41 | -96.1 | No | 2.65 | 22.5 | 27.5 | 4/25/2022 | 21.1 | 377 | Soybean | N/A | 110 | 8.48 | 151, 106 | Spring AA + at-plant UAN (45 kg N/ha, only in BAU) |
| NE02451 |  |  | X | X | 40.8 | -98 | Yes | 3.55 | 24 | 24 | 5/15/2022 | 22.1 | 418 | Soybean | N/A | 111 | 8.28 | 196, 151 | At-plant UAN |
| NE03451 |  | X | X | X | 40.9 | -97.6 | Yes | 3.4 | 20 | 31 | 5/17/2022 | 22 | 388 | Soybean | N/A | 110 | 7.92 | 189, 155 | Preplant MAP (11 kg N/ha) + at-plant UAN |
| NE04451 | X |  | X | X | 42.4 | -98 | Yes | N/A | N/A | N/A | 5/4/2022 | 20.2 | 383 | Soybean | DKC61-41RIB | 111 | 7.43 | 224, 179 | Preplant MAP, ATS + at-plant urea, AMS + side-dressed urea, AMS |
| SC01451 |  | X | X | X | 33.4 | -81.3 | No | 1.35 | 8 | 86 | 4/22/2022 | 24 | 467 | Cotton | N/A | 111 | 7.96 | 222, 177 | Preplant blend (43 kg N/ha) + side-dressed UAN |
| SD01451 |  | X | X | X | 44.5 | -96.7 | No | 4.1 | 22 | 43 | 5/6/2022 | 18.5 | 401 | Soybean | P0075AM | 100 | 8.76 | 196, 151 | At-plant urea |
| SD02451 |  | X | X | X | 44.3 | -96.7 | Yes | 3.55 | 24 | 41 | 5/4/2022 | 18.8 | 438 | Maize | P0075AM | 100 | 7.67 | 240, 195 | Preplant urea + at-plant urea (45 kg N/ha, only in BAU) |
| TX01451 |  |  | X | X | 35.1 | -101 | No | 3.1 | 38 | 28 | 5/31/2022 | 25.7 | 279 | Sorghum | DKC61-41RIB | 111 | 6.28 | 206, 165 | Preplant urea |
| WI01451 |  | X | X | X | 43 | -89.5 | No | 4.15 | 29 | 23 | 5/11/2022 | 18.4 | 534 | Maize | DKC52-18RIB | 102 | 7.78 | 194, 149 | Preplant UAN + at-plant UAN (45 kg N/ha, only in BAU) |

**Footnotes:**

^1^ “*x”* indicates that the measurements was collected at the trial

^2^ Soil samples taken for the top 15 cm of soil/

^3^ Seasonal estimates from planting to R6. Weather source: DAYMET.

^4^ First value corresponds to business-as-usual (BAU) and second value corresponds to the reduced N fertilizer treatments

^5^ BAU = Business-as-usual; UAN = urea-ammonium nitrate; AA = Anhydrous ammonia; MAP = Mono-ammonium phosphate, DAP = Diammonium phosphate; ATS = Ammonium Thiosulfate; AMS = ammonium sulfate.

| 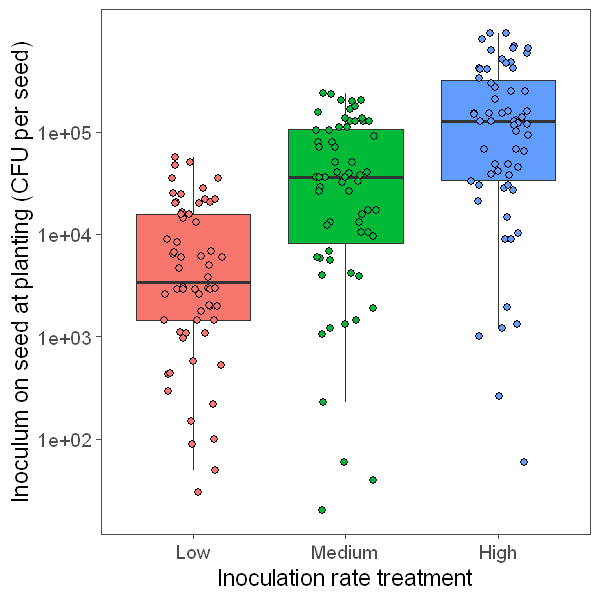 |
| --- |
| **Supplemental Figure S2. Estimated cell viability of the inoculum (Kv137-2253 + Ks6-5687) on seed at the time of planting (up to 45 days post inoculation) for the Low, Medium and High inoculation treatments in the inoculation rate trials.** |

**Supplemental Table S8. Estimated aboveground biomass N derived from the atmosphere (Ndfa) at flowering (VT-R1) in plots at the inoculation rate trials.**

| Year | Average |
| --- | --- |
| Aboveground biomass N  (kg N ha^-1^) | 191.2 (±8.06)^1^ |
| Ndfa (%) ^2^ | 11.1 (±5.68) |
| Ndfa (kg N ha^-1^) | 21.2 (±10.9)^3^ |

**Footnotes:**

^1^ Values within parentheses correspond to the standard error of the estimates.

^2^ Correspond to the average value of Medium and High inoculation rates

^3^Error for total Ndfa in kg N ha^-1^ was propagated using $y=(\hat{a}*\hat{b} )\sqrt[2]{\left( \frac{a_{SE}}{\hat{a}} \right)^{2}+\left( \frac{b_{SE}}{\hat{b}} \right)^{2}}$, where $\hat{a}$ and $\hat{b}$ are the estimates for aboveground biomass N N and Nfda (%), and $a_{SE}$ and $b_{SE}$ are their respective standard errors.

| 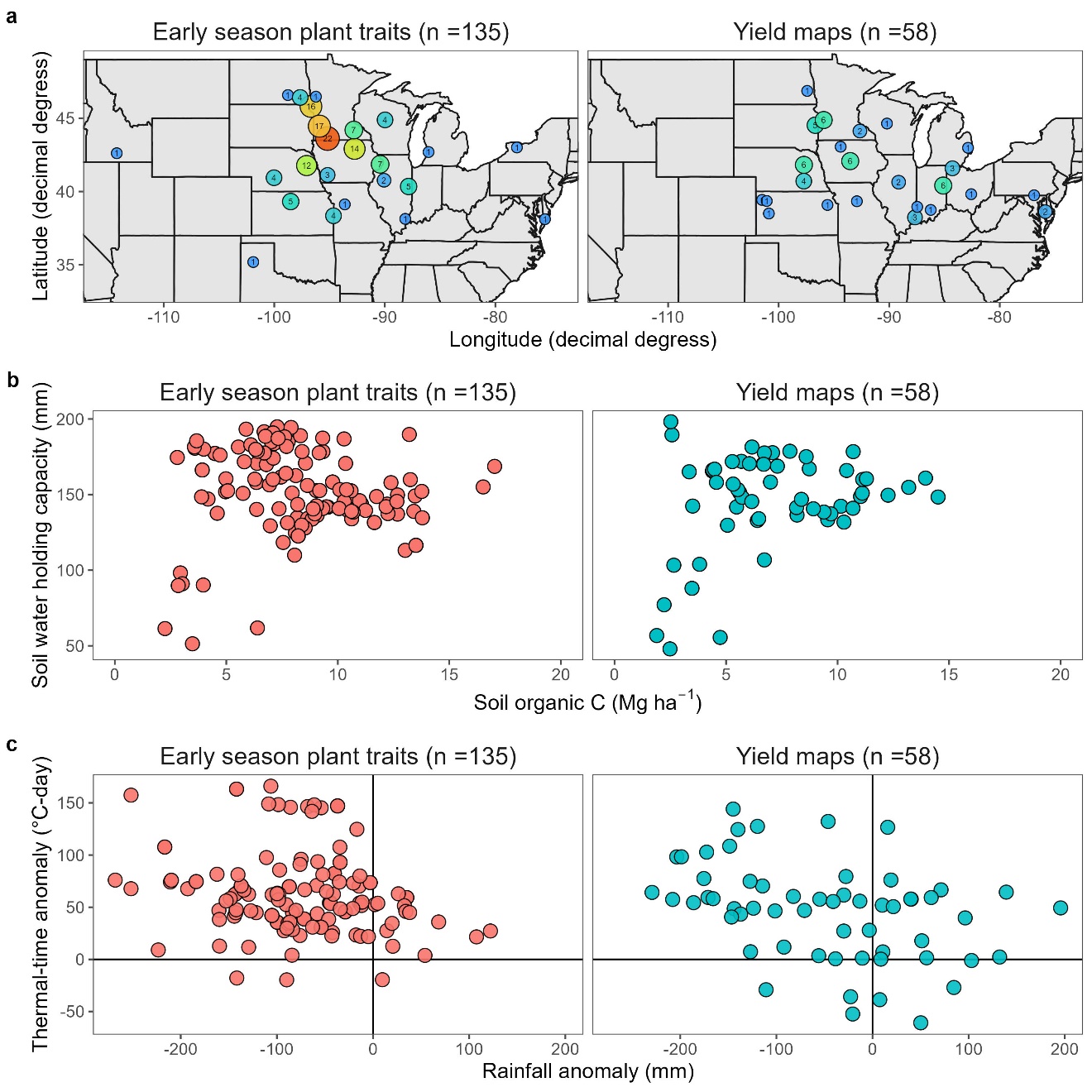 |
| --- |
| **Supplemental Figure S3**. Characterization of the grower fields from which early-season plant traits (2022, 2023) and yield maps (2021) were collected. **(a)** Geographical distribution of the field sites. Approximate locations are shown, aggregated to the closest quarter-decimal degree. **(b)** Soil characteristics to 1 m depth each site location, estimated from data available in the POLARIS dataset (Chaney et al. 2016). **(c)** Growing season (May-August) weather anomaly (versus the 30-year average) in rainfall and cumulative thermal time (°C-day; base temperature = 10 °C, maximum temperature - 30°C) for the trials. Weather source (Iowa Environmental Mesonet Reanalysis; <https://mesonet.agron.iastate.edu/iemre/>). |

| 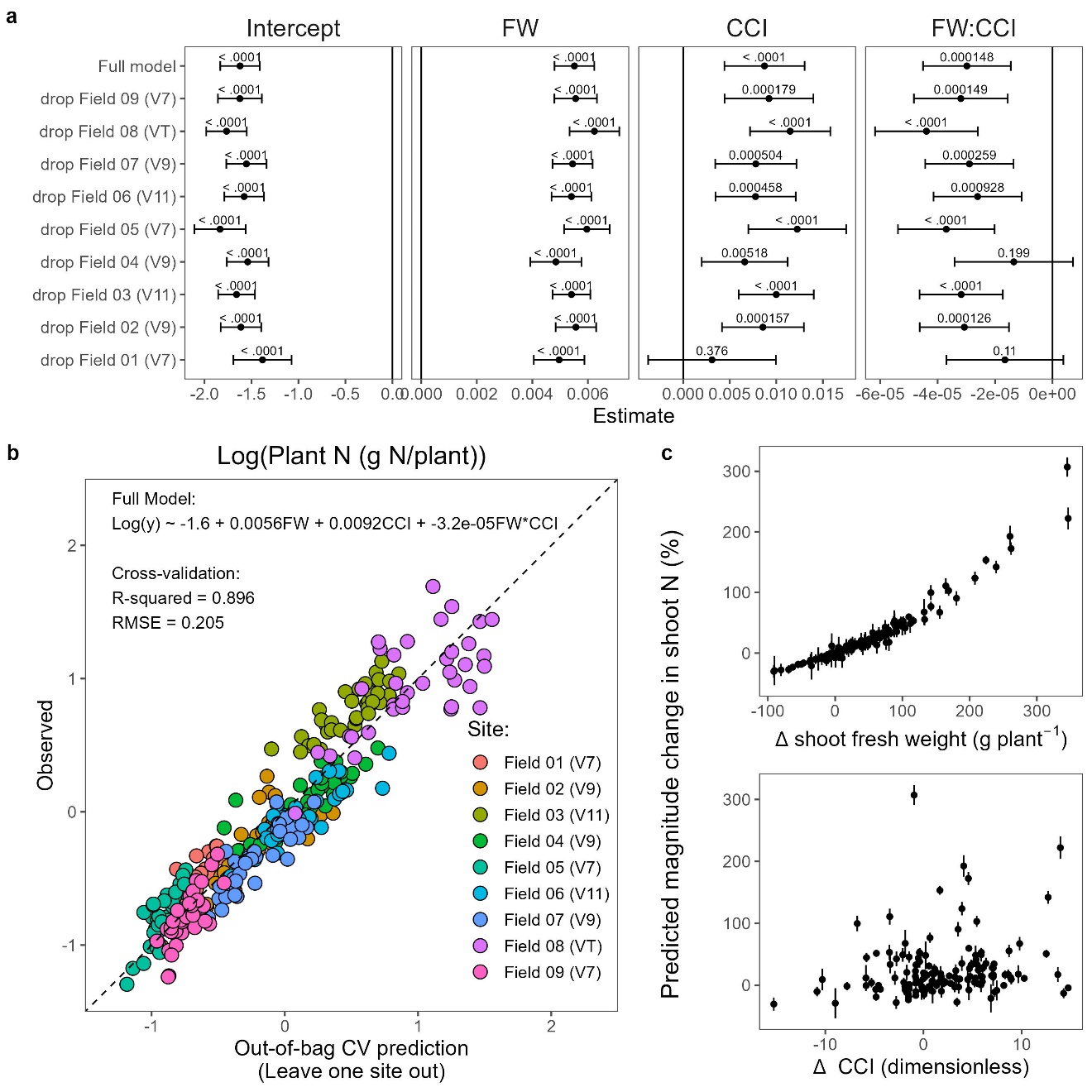 |
| --- |
| **Supplemental Figure S4**. **Developing a model for predicting magnitude changes in maize shoot N during the vegetative stages from shoot fresh weight (FW) and leaf chlorophyl concentration index (CCI).** **(a)** Multiple regression coefficient estimates for full and cross-validated models. Error bars show 95% confidence intervals, and number labels indicate p-value of the estimate. **(b)** Performance of the cross-validated models when predicting out-of-bag (i.e., unseen) data. **(c)** Predicted changes in the magnitude of total shoot N as driven by the changes in FW and CCI observed between the business-as-usual and PROVEN 40 inoculated treatments at the on-farm grower trials (n = 165). |

| 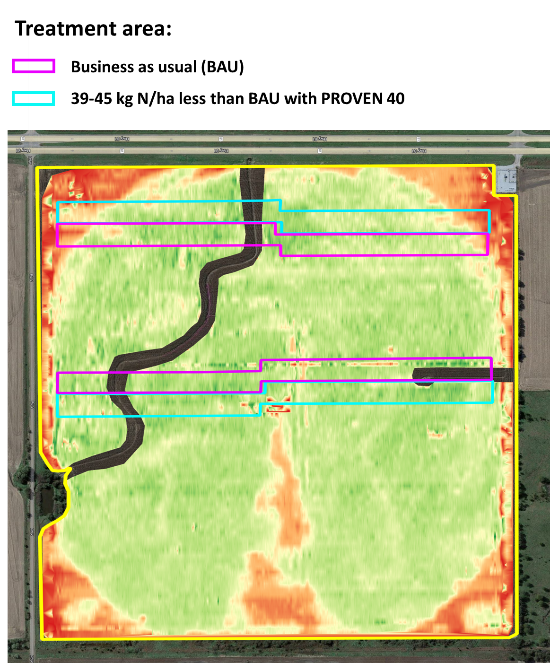 |
| --- |
| **Supplemental Figure S5**. **Example of one of the 58 yield monitor maps provided by cooperator growers in 2021.** Growers applied the treatment (39-45 kg N ha^-1^ less fertilizer than BAU with PROVEN 40) across one or more strips in their fields. The size and number the strips varied among locations, with an average an area of 11.3 ha of treated area by location. For the BAU treatment area, we selected an equally sized area adjacent to each strip. This aimed to represent a side-by-side comparison at each location. |

**Supplemental Table S9.** Summary of linear mixed-effect model fitting for field δ15N datasets.

| **Dataset** | **Tissue & timing** | **Site-Year** | **Model**  **(LMER notation)^1^** | **Comments** |
| --- | --- | --- | --- | --- |
| Pivot Bio, Inc.  Natural Abundance | Aboveground biomass @ VT-R1 | 16 | log(y) ~ trt + (1\|sy/rep) | - Used log(y) to control for heteroskedasticity.  -Weights determined based on the median δ15N of the uninoculated control |
| Purdue University  ACRE Farm  Natural Abundance | Leaf tips @ V8 | 2021, 2022 | y ~ trt*sy + nr + (1\|sy:rep:nr) | - Cross-product random effect due to singular fit under nested (1\|sy/rep/nr). |
|  | Aboveground biomass @ V8, R1 and R6 | 2021 | log(y) ~ gs*trt + gs*nr + (1\|rep:nr) | - Used log(y) to control for heteroskedasticity.  - Reduced model because three-way interaction (full model) was not significant and had higher AIC.  - Cross-product random effect due to singular fit under nested (1\|rep/nr). |
|  | Aboveground biomass @ V8, R1 and R6 | 2022 | log(y) ~ gs*trt + gs*nr + (1\|rep/nr) | - Used log(y) to control for heteroskedasticity.  - Reduced model because three-way interaction (full model) was not significant and had higher AIC. |
| University of Wisconsin-Madison  Enrichment- Dilution | Leaf tips @ V4-VT | Hancock 2021 | log(y) ~ trt + gs + (1\|rep/plot) | - Used log(y) to control for heteroskedasticity.  - Reduced model because two-way interaction (full model) was not significant and had higher AIC.  -  - |
|  | Leaf tips @ V4-VT | Hancock 2022 | log(y) ~ trt + gs + (1\|rep/plot) |  |
|  | Leaf tips @ V4-VT | Arlington 2021 | log(y) ~ trt + gs + (1\|rep/plot) |  |

**Footnotes:**

^1^ Model notation follows the linear mixed-effect regression lmer() function in the lme4 library in R. Fixed effects: trt = treatment; sy = site year; nr = nitrogen fertilizer rate; gs = crop growth stage. Random effects: (1|sy/rep) = replicate within site year; (1|sy:rep:nr) = random intercept at each cross-product of nitrogen rate, replicate and site year (split plot); (1|rep:nr) = random intercept at each cross-product of nitrogen rate and replication (split plot); (1|rep/nr) = random intercept at each nitrogen rate within replication (split plot); and (1|rep/nr) = random intercept at each plot within replication (repeated measures).

## Supplemental information references

Bloch S E, Clark R, Gottlieb S S, Kent Wood L, Shah N, Mak S M, Lorigan J G, Johnson J, Davis-Richardson A G, Williams L, McKellar M, Soriano D, Petersen M, Horton A, Smith O, Wu L, Tung E, Broglie R, Tamsir A and Temme K 2020 Biological nitrogen fixation in maize: Optimizing nitrogenase expression in a root-associated diazotroph *J Exp Bot* **71** 4591–603

Chaney N W, Minasny B, Herman J D, Nauman T W, Brungard C W, Morgan C L S, McBratney A B, Wood E F and Yimam Y 2019 POLARIS soil properties: 30-m probabilistic maps of soil properties over the contiguous United States *Water Resour Res* **55** 2916–38 Online: https://onlinelibrary.wiley.com/doi/full/10.1029/2018WR022797

Francis H R, Ma T F and Ruark M D 2021 Toward a standardized statistical methodology comparing optimum nitrogen rates among management practices: A bootstrapping approach *Agricultural & Environmental Letters* **6** e20045 Online: https://onlinelibrary.wiley.com/doi/full/10.1002/ael2.20045

Selvaraj G and Iyer V N 1983 Suicide plasmid vehicles for insertion mutagenesis in Rhizobium meliloti and related bacteria. *J Bacteriol* **156** 1292 Online: /pmc/articles/PMC217980/?report=abstract

Thornton M M, Shrestha R, Wei Y, Thornton P E, Kao S-C and Wilson B E 2022 Daymet: Annual Climate Summaries on a 1-km Grid for North America, Version 4 R1 *ORNL DAAC, Oak Ridge, Tennessee, USA. https://doi.org/10.3334/ORNLDAAC/2130*

Wen A, Havens K L, Bloch S E, Shah N, Higgins D A, Davis-Richardson A G, Sharon J, Rezaei F, Mohiti-Asli M, Johnson A, Abud G, Ane J M, Maeda J, Infante V, Gottlieb S S, Lorigan J G, Williams L, Horton A, McKellar M, Soriano D, Caron Z, Elzinga H, Graham A, Clark R, Mak S M, Stupin L, Robinson A, Hubbard N, Broglie R, Tamsir A and Temme K 2021 Enabling biological nitrogen fixation for cereal crops in fertilized fields *ACS Synth Biol* **10** 3264–77

## R packages used (including dependencies)

R version 4.1.3 (2022-03-10)

Platform: x86_64-w64-mingw32/x64 (64-bit)

Running under: Windows 10 x64 (build 22621)

Matrix products: default

locale:

[1] LC_COLLATE=English_United States.1252

[2] LC_CTYPE=English_United States.1252

[3] LC_MONETARY=English_United States.1252

[4] LC_NUMERIC=C

[5] LC_TIME=English_United States.1252

attached base packages:

[1] stats graphics grDevices utils datasets methods base

other attached packages:

[1] ggthemes_4.2.4 png_0.1-8 scales_1.2.1 ggpubr_0.6.0

[5] multcomp_1.4-23 TH.data_1.1-2 MASS_7.3-58.3 survival_3.5-7

[9] mvtnorm_1.2-3 emmeans_1.8.6 caret_6.0-94 lattice_0.21-8

[13] DHARMa_0.4.6 car_3.1-2 carData_3.0-5 glmmTMB_1.1.8

[17] lmerTest_3.1-3 lme4_1.1-34 Matrix_1.6-2 nlme_3.1-163

[21] foreach_1.5.2 broom_1.0.5 readxl_1.4.3 lubridate_1.9.2

[25] forcats_1.0.0 stringr_1.5.0 dplyr_1.1.2 purrr_1.0.2

[29] readr_2.1.4 tidyr_1.3.0 tibble_3.2.1 ggplot2_3.4.2

[33] tidyverse_2.0.0

## R packages Citations

R Core Team (2022). *R: A Language and Environment for Statistical. Computing*. R Foundation for Statistical Computing, Vienna, Austria. https://www.R-project.org

Arnold J (2021). *ggthemes: Extra Themes, Scales and Geoms for 'ggplot2'*. R package version 4.2.4, https://CRAN.R-project.org/package=ggthemes

Urbanek S (2022). *png: Read and write PNG images*. R package version 0.1-8, https://CRAN.R-project.org/package=png

Wickham H, Seidel D (2022). *scales: Scale Functions for Visualization*. R package version 1.2.1, https://CRAN.R-project.org/package=scales.

Kassambara A (2023). *ggpubr: 'ggplot2' Based Publication Ready Plots*. R package version 0.6.0, <URL:

https://CRAN.R-project.org/package=ggpubr.

Hothorn T, Bretz F, Westfall P (2008). "Simultaneous Inference in General Parametric Models." *Biometrical Journal*, **50**(3), 346-363.

Hothorn T (2023). *TH.data: TH's Data Archive*. R package version 1.1-2, https://CRAN.R-project.org/package=TH.data.

Venables WN, Ripley BD (2002). *Modern Applied Statistics with S*,Fourth edition. Springer, New York. ISBN 0-387-95457-0, https://www.stats.ox.ac.uk/pub/MASS4/

Therneau T (2023). *A Package for Survival Analysis in R*. R package version 3.5-7, https://CRAN.R-project.org/package=survival

Terry M. Therneau, Patricia M. Grambsch (2000). *Modeling Survival Data: Extending the Cox Model* Springer, New York. ISBN 0-387-98784-3.

Genz A, Bretz F (2009). *Computation of Multivariate Normal and t Probabilities*, series Lecture Notes in Statistics. Springer-Verlag, Heidelberg. ISBN 978-3-642-01688-2.

Lenth R (2023). *emmeans: Estimated Marginal Means, aka Least-Squares Means*. R package version 1.8.6, https://CRAN.R-project.org/package=emmeans.

Hartig F (2022). *DHARMa: Residual Diagnostics for Hierarchical (Multi-Level / Mixed) Regression Models*. R package version 0.4.6, https://CRAN.R-project.org/package=DHARMa

Fox J, Weisberg S (2019). *car: An R Companion to Applied Regression*, Third edition. Sage, Thousand Oaks CA. https://socialsciences.mcmaster.ca/jfox/Books/Companion/

Fox J, Weisberg S, Price B (2022). *carData: Companion to Applied Regression Data Sets*. R package version 3.0-5, https://CRAN.R-project.org/package=carData

Kuhn, Max (2008). "Building Predictive Models in R Using the caret Package." *Journal of Statistical Software*, *28*(5), 1 26. https://www.jstatsoft.org/index.php/jss/article/view/v028i05

Sarkar D (2008). *Lattice: Multivariate Data Visualization with R*. Springer, New York. ISBN 978-0-387-75968-5, http://lmdvr.r-forge.r-project.org

Brooks ME, Kristensen K, van Benthem KJ, Magnusson A, Berg CW, Nielsen A, Skaug HJ, Maechler M, Bolker BM (2017). "glmmTMB Balances Speed and Flexibility Among Packages for Zero-inflated Generalized Linear Mixed Modeling." *The R Journal*, *9*(2), 378-400 https://doi.org/10.32614/RJ-2017-066

Kuznetsova A, Brockhoff PB, Christensen RHB (2017). "lmerTest Package: Tests in Linear Mixed Effects Models." Journal of Statistical Software, *82*(13), 1-26. https://doi.org/10.18637/jss.v082.i13

Bates D, M chler M, Bolker B, Walker S (2015). "Fitting Linear Mixed-Effects Models Using lme4." *Journal of Statistical Software*, *67*(1), 1-48. doi: 10.18637/jss.v067.i01 https://doi.org/10.18637/jss.v067.i01

Bates D, Maechler M, Jagan M (2023). *Matrix: Sparse and Dense Matrix Classes and Methods*. R package version 1.6-2, https://CRAN.R-project.org/package=Matrix

Pinheiro J, Bates D, R Core Team (2023). *nlme: Linear and Nonlinear Mixed Effects Models*. R package version 3.1-163, https://CRAN.R-project.org/package=nlme.

Pinheiro JC, Bates DM (2000). *Mixed-Effects Models in S and S-PLUS*. Springer, New York. https://doi.org/10.1007/b98882.

Microsoft, Weston S (2022). *foreach: Provides Foreach Looping Construct*. R package version 1.5.2, https://CRAN.R-project.org/package=foreach

Robinson D, Hayes A, Couch S (2023). *broom: Convert Statistical Objects into Tidy Tibbles*. R package version 1.0.5, https://CRAN.R-project.org/package=broom.

Wickham H, Bryan J (2023). *readxl: Read Excel Files*. R package version 1.4.3, https://CRAN.R-project.org/package=readxl.

Grolemund G, Wickham H (2011). "Dates and Times Made Easy with lubridate." *Journal of Statistical Software*, **40**(3), 1-25. https://www.jstatsoft.org/v40/i03/.

Wickham H (2023). *forcats: Tools for Working with Categorical Variables (Factors)*. R package version 1.0.0, https://CRAN.R-project.org/package=forcats

Wickham H (2022). *stringr: Simple, Consistent Wrappers for Common String Operations*. R package version 1.5.0, https://CRAN.R-project.org/package=stringr.

Wickham H, Francois R, Henry L, M ller K, Vaughan D (2023). *dplyr: A Grammar of Data Manipulation*. R package version 1.1.2, https://CRAN.R-project.org/package=dplyr>.

Wickham H, Henry L (2023). *purrr: Functional Programming Tools*. R package version 1.0.2, <URL: https://CRAN.R-project.org/package=purrr>.

Wickham H, Hester J, Bryan J (2023). *readr: Read Rectangular Text Data*. R package version 2.1.4, https://CRAN.R-project.org/package=readr.

Wickham H, Vaughan D, Girlich M (2023). *tidyr: Tidy Messy Data*. R package version 1.3.0, <URL: https://CRAN.R-project.org/package=tidyr

Mueller K, Wickham H (2023). t*ibble: Simple Data Frames*. R package version 3.2.1, <URL: https://CRAN.R-project.org/package=tibble.

Wickham H (2016). *ggplot2: Elegant Graphics for Data Analysis*. Springer-Verlag New York. ISBN 978-3-319-24277-4, https://ggplot2.tidyverse.org

Wickham H, Averick M, Bryan J, Chang W, McGowan LD, Francois R, Grolemund G, Hayes A, Henry L, Hester J, Kuhn M, Pedersen TL, Miller E, Bache SM, Mueller K, Ooms J, Robinson D, Seidel DP, Spinu V, Takahashi K, Vaughan D, Wilke C, Woo K, Yutani H (2019). "Welcome to the tidyverse." *Journal of Open Source Software*, **4**(43), 1686. https://doi.org/10.21105/joss.01686
